# A Deterministic Model Predicts the Properties of Stochastic Calcium Oscillations in Airway Smooth Muscle Cells

**DOI:** 10.1371/journal.pcbi.1003783

**Published:** 2014-08-14

**Authors:** Pengxing Cao, Xiahui Tan, Graham Donovan, Michael J. Sanderson, James Sneyd

**Affiliations:** 1 Department of Mathematics, University of Auckland, Auckland, New Zealand; 2 Department of Microbiology and Physiological Systems, University of Massachusetts Medical School, Worcester, Massachusetts, United States of America; University of California, San Diego, United States of America

## Abstract

The inositol trisphosphate receptor (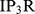) is one of the most important cellular components responsible for oscillations in the cytoplasmic calcium concentration. Over the past decade, two major questions about the 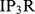 have arisen. Firstly, how best should the 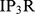 be modeled? In other words, what fundamental properties of the 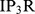 allow it to perform its function, and what are their quantitative properties? Secondly, although calcium oscillations are caused by the stochastic opening and closing of small numbers of 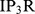, is it possible for a deterministic model to be a reliable predictor of calcium behavior? Here, we answer these two questions, using airway smooth muscle cells (ASMC) as a specific example. Firstly, we show that periodic calcium waves in ASMC, as well as the statistics of calcium puffs in other cell types, can be quantitatively reproduced by a two-state model of the 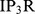, and thus the behavior of the 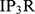 is essentially determined by its modal structure. The structure within each mode is irrelevant for function. Secondly, we show that, although calcium waves in ASMC are generated by a stochastic mechanism, 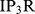 stochasticity is not essential for a qualitative prediction of how oscillation frequency depends on model parameters, and thus deterministic 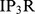 models demonstrate the same level of predictive capability as do stochastic models. We conclude that, firstly, calcium dynamics can be accurately modeled using simplified 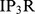 models, and, secondly, to obtain qualitative predictions of how oscillation frequency depends on parameters it is sufficient to use a deterministic model.

## Author Summary

The inositol trisphosphate receptor () is one of the most important cellular components responsible for calcium oscillations. Over the past decade, two major questions about the  have arisen. Firstly, what fundamental properties of the  allow it to perform its function? Secondly, although calcium oscillations are caused by the stochastic properties of small numbers of  is it possible for a deterministic model to be a reliable predictor of calcium dynamics? Using airway smooth muscle cells as an example, we show that calcium dynamics can be accurately modeled using simplified  models, and, secondly, that deterministic models are qualitatively accurate predictors of calcium dynamics. These results are important for the study of calcium dynamics in many cell types.

## Introduction

Oscillations in cytoplasmic calcium concentration (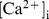), mediated by inositol trisphosphate receptors (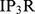; a calcium channel that releases calcium ions (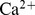) from the endoplasmic or sarcoplasmic reticulum (ER or SR) in the presence of inositol trisphosphate (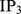)) play an important role in cellular function in many cell types. Hence, a thorough knowledge of the behavior of the 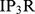 is a necessary prerequisite for an understanding of intracellular 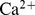 oscillations and waves. Mathematical and computational models of the 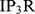 play a vital role in studies of 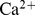 dynamics. However, over the past decade, two major questions about 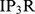 models have arisen.

Firstly, how best should the 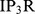 be modeled? Models of the 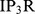 have a long history, beginning with the heuristic models of [1]–[3]. With the recent appearance of single-channel data from 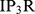 *in vivo* [4], [5], a new generation of Markov 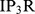 models has recently appeared [6], [7]. These models show that 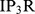 exist in different modes with different open probabilities. Within each mode there are multiple states, some open, some closed. Importantly, it was found [8] that time-dependent transitions between different modes are crucial for reproducing 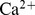 puff data from [9]. However, it is not yet clear whether transitions between states within each mode are important, or whether all the important behaviors are captured simply by inter-mode transitions.

Secondly, why do deterministic models of the 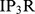 perform so well as predictive models? Deterministic models of the 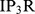 have proven to be useful predictive models in a range of cell types. For example, 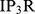-based models have been developed to study 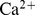 oscillations in airway smooth muscle cells (ASMC) [10]–[13], and these models have made predictions which have been confirmed experimentally. This shows the usefulness of such models in advancing our understanding of how intracellular 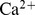 oscillations and waves are initiated and controlled in ASMC. However, these models are deterministic models which assume infinitely many 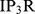 per unit cell volume, an assumption that contradicts experimental findings in many cell types showing that 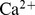 puffs and spikes occur stochastically, and that intracellular 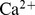 waves and oscillations arise as an emergent property of fundamental stochastic events [9], [14], [15].

Here, we answer these two fundamental modeling questions using data and models from ASMC. Firstly, we show that a simple model of the 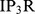, involving only two states with time-dependent transitions, suffices to generate correct dynamics of 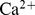 puffs and oscillations. Secondly, we show that, although 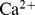 oscillations in ASMC are generated by a stochastic mechanism, a deterministic model can make the same qualitative predictions as the analogous stochastic model, indicating that deterministic models, that require much less computational time and complexity, can be used to make reliable predictions. Although we work in the specific context of ASMC, our results are applicable to other cell types that exhibit similar 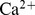 oscillations and waves.

## Results

### A two-state model of the 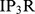 is sufficient to reproduce function

**Figure 1:**
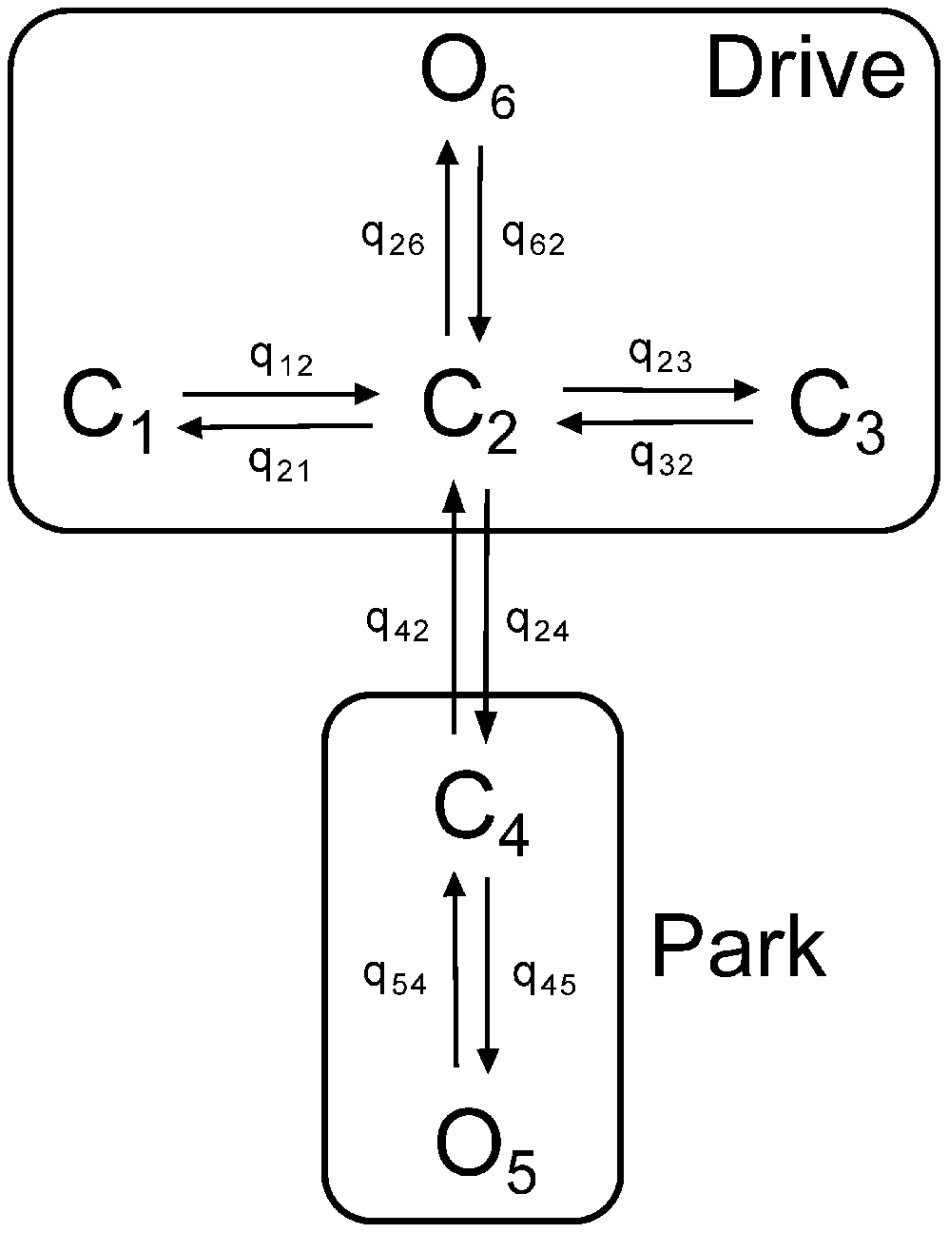
The structure of the Siekmann 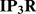 model. The 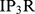 model is comprised of two modes. One is the drive mode containing three closed states 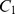, 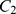, 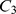 and one open state 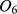. The other is the park mode which includes one closed state 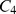 and one open state 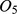. 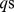 are rates of state-transitions between two adjacent states and 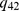 and 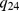 are transitions between the two modes [7].

We have previously shown [8] that the statistics of 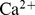 puffs in SH-SY5Y cells can be reproduced by a Markov model of the 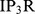 based on the steady-state data of [5] and the time-dependent data of [4]. In this model the 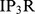 can exist in 6 different states, grouped into two modes, which we call *Drive* and *Park* (see Fig. 1). The Drive mode (which contains 4 states; 1 open and 3 closed) has an average open probability of around 0.7, while the Park mode (which contains the remaining two states; 1 open and 1 closed) has an open probability close to zero. Transitions between states within each mode are independent of 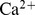 and 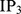; only the transitions between modes are ligand-dependent.

**Figure 2:**
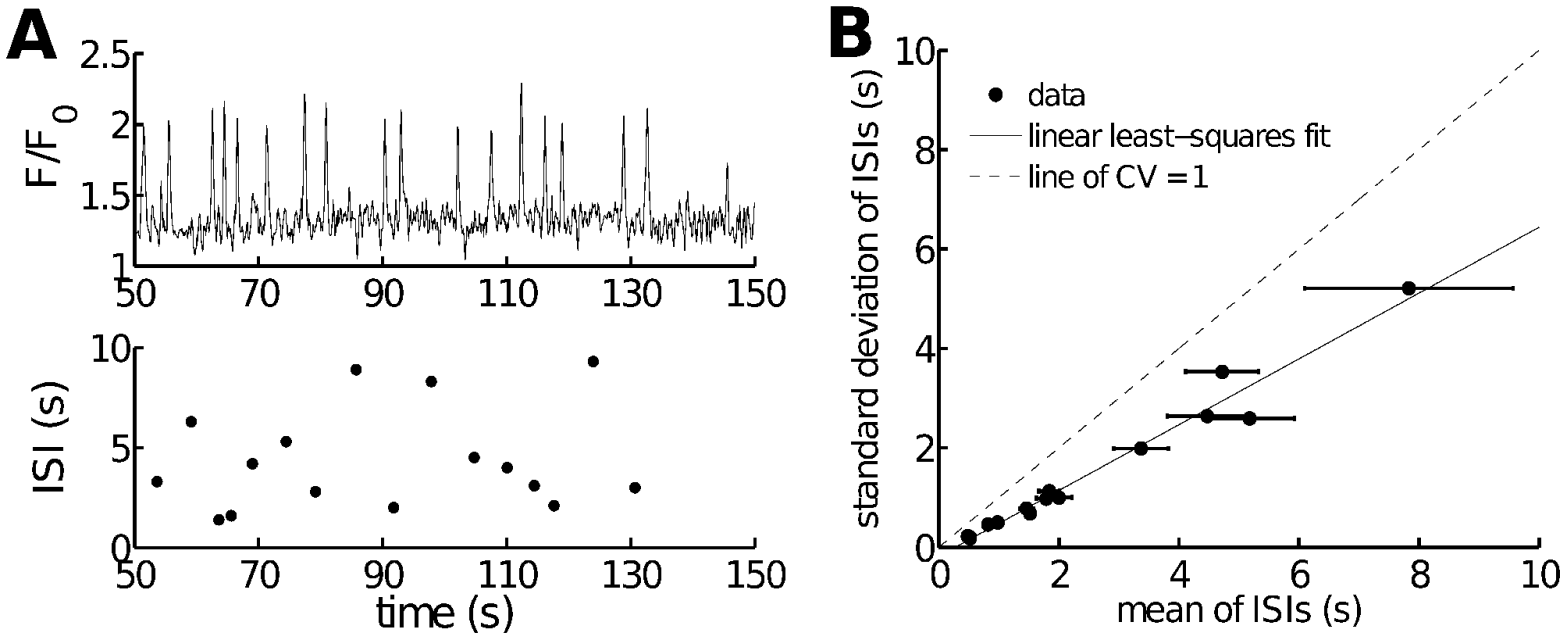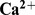 oscillations in ASMC in lung slices are generated by a stochastic mechanism. **A**: experimental 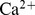 spiking in ASMC in lung slices, stimulated with 50 nM MCh. In the upper panel we filter out baseline noise by using a low threshold of 1.42 (relative fluorescence intensity) and then choose samples with amplitude larger than 1.75. The ISI calculated from the upper panel is shown in the lower panel. **B**: relationship between the standard deviation and the mean of experimental ISIs. Data obtained from 14 ASMC in 5 mouse lung slices. The relationship is approximately linear with a slope of 0.66, which implies that an inhomogeneous Poisson process governs the generation of oscillations. The dashed line indicates where the coefficient of variation (CV) is 1 (as it is for a pure Poisson process). Variation in ISI is mainly caused by both use of different doses of MCh and different sensitivities of different cells to MCh. Error bars indicate the standard errors of the means (SEM).

In our previous study on calcium puffs [8], we showed that, to reproduce the experimentally observed non-exponential interspike interval (ISI) distribution and coefficient of variation (CV) of ISI smaller than 1, the time-dependent intermodal transitions are crucial. Lack of time dependencies in the Siekmann model leads to exponential ISI distributions and CV = 1, which is not the case for calcium spikes in ASMC. Fig. 2A shows an example of 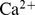 oscillations generated by 50 nM methacholine (MCh, an agonist that can induce the production of 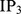 by binding to a G protein-coupled receptor in the cell membrane) in ASMC. By gathering data from 14 cells in 5 mouse lung slices, we found that the standard deviation of the interspike interval (ISI) is approximately a linear function of the ISI mean, with a slope clearly between 0 and 1 (i.e. 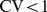), indicating that the spikes are generated by an inhomogeneous Poisson process (a slope of 1 would denote a pure Poisson process) (see Fig. 2B). This shows the necessity of inclusion of time-dependent transitions for mode-switching.

**Figure 3:**
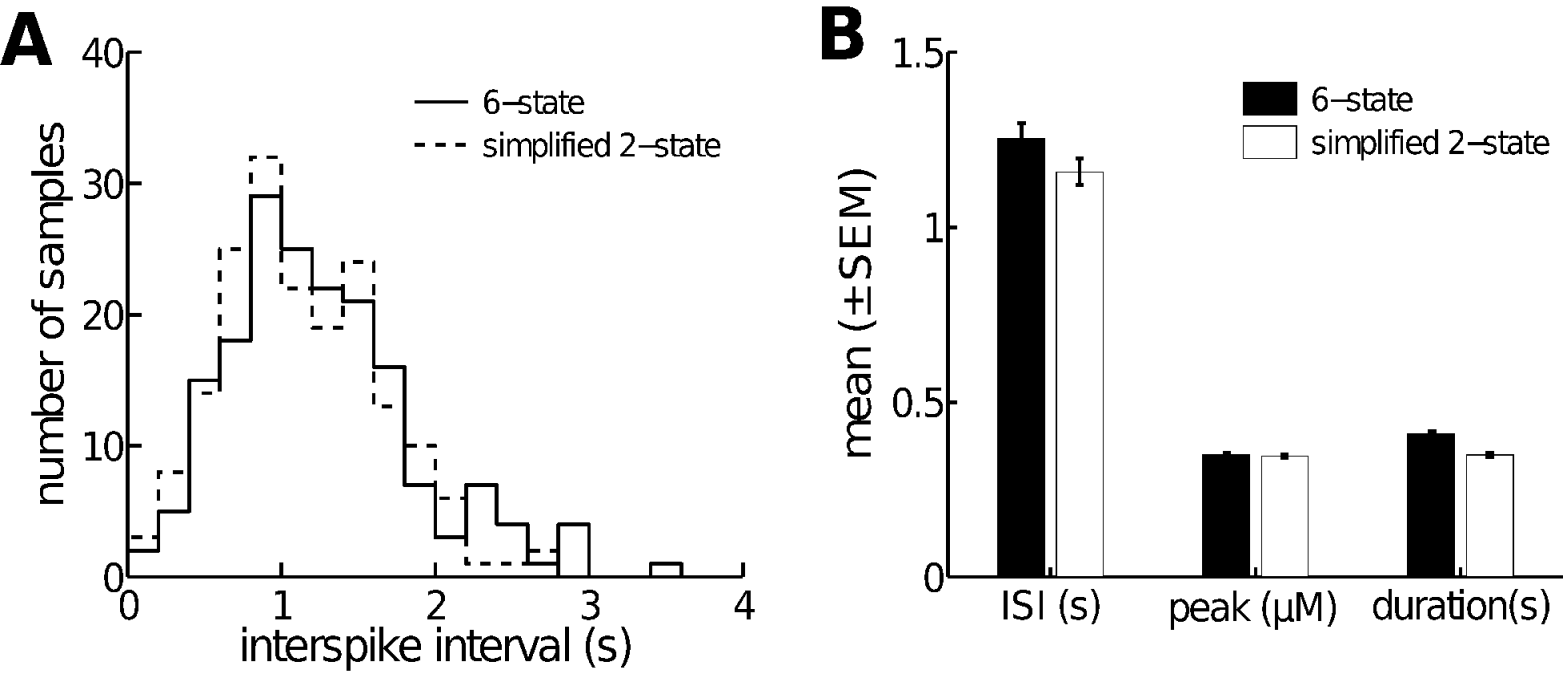
A 2-state open/closed model quantitatively reproduces the 6-state 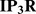 model. **A**: histograms of interspike interval (ISI) distribution for both the 6-state and the simplified models. The ISI is defined to be the waiting time between successive spikes. Each histogram contain an equal number of samples (180). **B**: comparison of average ISI, average peak value of 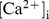 (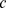 in the model) and average spike duration. All distributions were computed at a constant 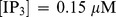.

**Figure 4:**
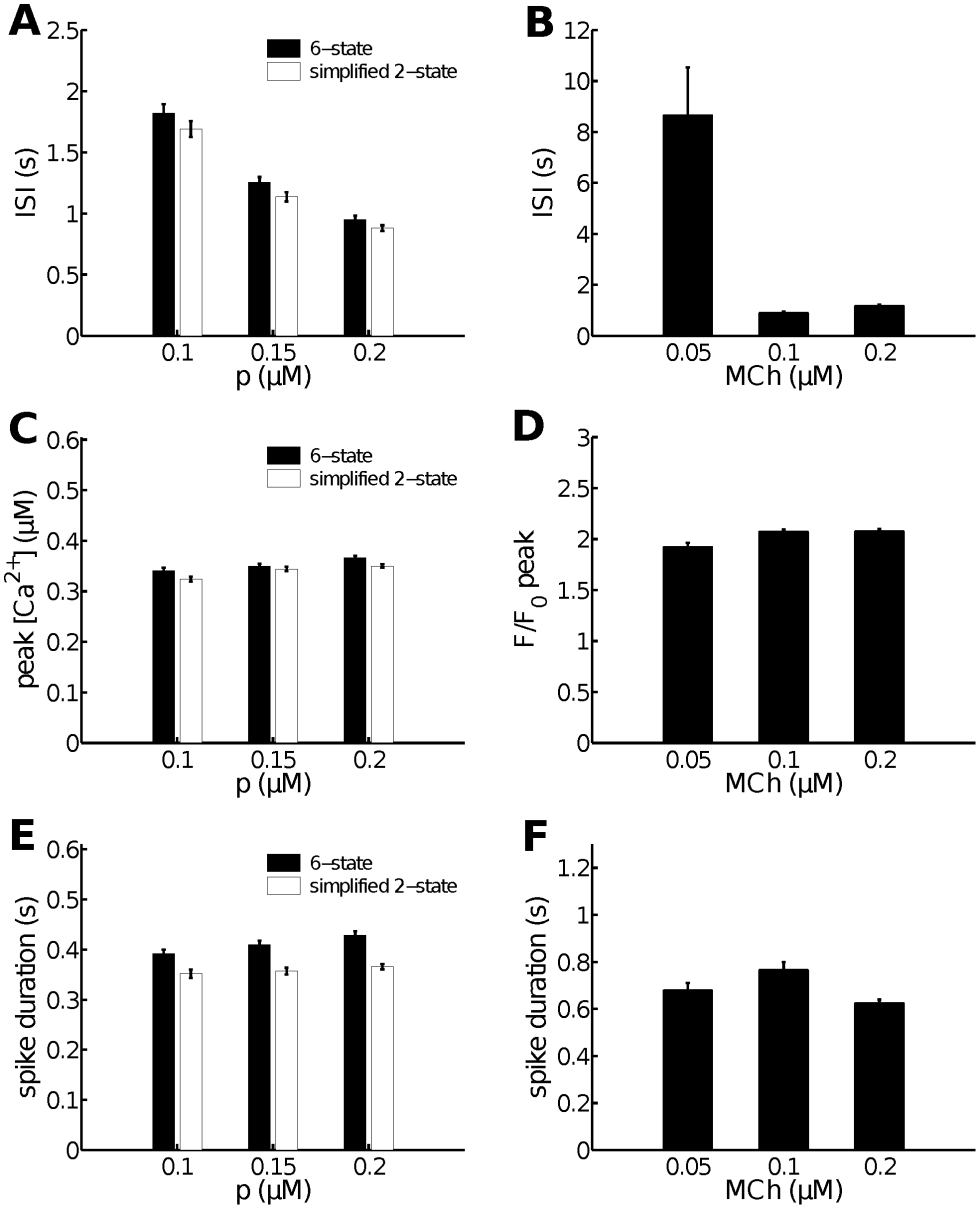
More detailed comparisons between the 2-state and the 6-state 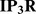 models, and a comparison to experimental data. As a function of 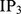 concentration (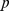), the two models give the same ISI (**A**), peak 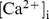 (**C**) and spike duration (**E**). These results agree qualitatively with experimental data, as shown in panels **B**, **D** and **F** respectively. Quantitative comparisons are generally not possible as the relationship between 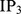 concentration and agonist concentration is not known. Error bars represent 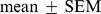. Data for each MCh concentration are obtained from at least three different cells from at least two different lung slices.

Using a quasi-steady-state approximation, and ignoring states with very low dwell times, it is possible to construct a simplified two-state version of the full six-state model (see *Materials and Methods*). In the simplified model the intramodal structure is ignored, and only the intermodal transitions have an effect on 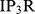 behavior. In Fig. 3 we compared the simplified 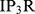 model to the full six-state model. Both models have the same distribution of interspike interval, spike amplitude and spike duration. Moreover, by looking at a more detailed comparison between the two model results (Figs. 4A, C and E) and experimental data (Figs. 4B, D and F), we found the 2-state model not only can reproduce the behaviour of the 6-state model, but can also qualitatively reproduce experimental data. The average experimental ISI shows a clear decreasing trend as MCh concentration increases (although a saturation occurs in the data for high MCh), a trend that is mirrored by the model results as the 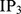 concentration increases. Unfortunately, since the exact relationship between MCh concentration and 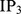 concentration is uncertain, a quantitative comparison is not possible. In both model and experimental results, the average peak and duration of the oscillations are nearly independent of agonist concentration. The quantitative difference in spike duration between the model results and the data in Figs. 4E and F are most likely due to choice of calcium buffering parameters. For example, adding 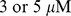 fast 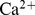 buffer (see *Materials and Methods*) increases the average spike duration to 0.54 s or 0.7 s respectively, which are close to the levels shown in the data.

Thus, the intramodal structure of the six-state model is essentially unimportant, as the model behavior (in terms of the statistics of puffs and oscillations) is governed almost entirely by the time dependence of the intermode transitions, particularly the time dependence of the rapid inhibition of the 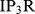 by high 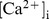, and the slow recovery from inhibition by 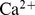. The multiple states within each mode are necessary to obtain an acceptable quantitative fit to single-channel data, but are nevertheless of limited importance for function. Hence, even when simulating microscopic events such as 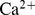 puffs it is sufficient to use a simpler, faster, two-state model, rather than a more complex six-state model. In the following, we will use the 2-state 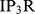 model to generate all the simulation results.

### Prediction of stochastic 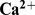 behavior by a deterministic model

Although the data (Fig. 2) show that 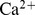 oscillations in ASMC are generated by a stochastic process, not a deterministic one, we wish to know to what extent a deterministic model can be used to make qualitative (and experimentally testable) predictions. Our simplified 2-state Markov model of the 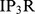 can be converted to a deterministic model (see *Materials and Methods*). The result is a system of ordinary differential equations (ODEs) with four variables, which takes into account the increased 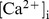 at an open 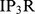 pore, as well as the increased 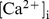 within a cluster of 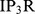; the four variables are the 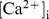 outside the 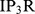 cluster (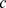), the 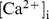 within the 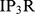 cluster (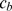), the total intracellular 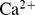 concentration (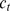) and an 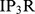 gating variable (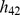). We refer to the reduced 4D model as the deterministic model for all the results and analyses.

Note that there is no physical or geometric constraint enforcing a high local 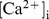; in this case the spatial heterogeneity arises solely from the low diffusion coefficient of 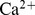. Our use of 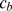 is merely a highly simplified way of introducing spatial heterogeneity of the 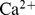 concentration. Since the 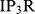 can only “see” 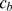 (as well as the 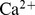 concentration right at the mouth of an open channel, which we denote by 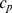), but cannot be influenced directly by 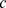 (the experimentally observed 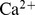 signal), our approach allows for the functional differentiation of the rapid local oscillatory 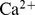 in the cluster, from the slower 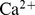 signal in the cytoplasm, without the need for computationally intensive simulations of a partial differential equation model. Quantitative accuracy is thus sacrificed for computational convenience.

**Figure 5:**
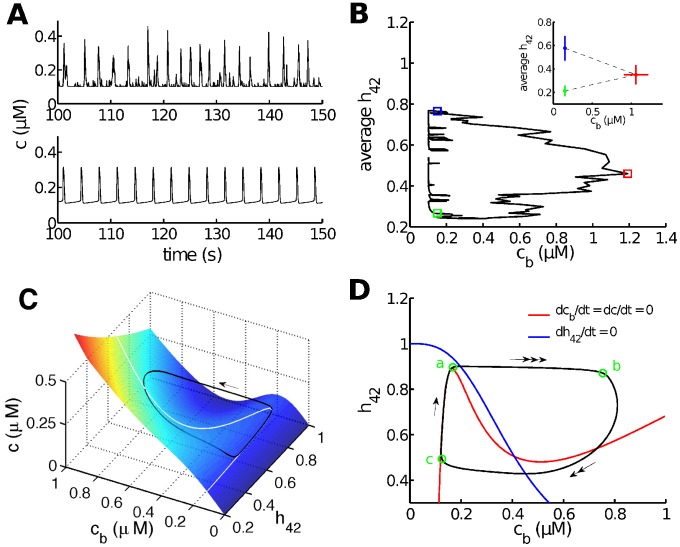
Stochastic and deterministic simulations exhibit similar dynamic properties. **A**: simulated stochastic (upper panel) or deterministic (lower panel) 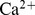 oscillations at 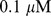 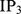. **B**: a typical stochastic solution projected on the 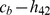 plane. The average 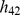 represents the average value of 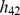 over the 20 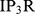. Statistics (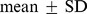) of the initiation point (blue square), the peak (red square) and termination point (green square) are shown in the inset. 116 samples are obtained by applying a low threshold of 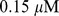 and a high threshold of 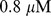 to 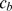. **C**: a typical periodic solution of the deterministic model (black curve), plotted in the 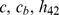 phase space. The arrow indicates the direction of movement. 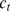 is the slowest variable so that its variation during an oscillation is very small. This allows to treat 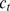 as a constant (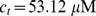 in this case) and study the dynamics of the model in the 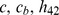 phase space. The color surface is the surface where 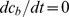 (called the critical manifold). The white N-shaped curve is the intersection of the critical manifold and the surface 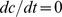. **D**: projection of the periodic solution to the 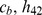 plane. The red N-shaped curve is the projection to the 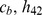 plane of the white curve shown in **C**. The evolution of the deterministic solution exhibits three different time scales separated by green circles (labelled by a, b and c) and indicated by arrows (triple arrow: fastest; double arrow: intermediate; single arrow: slowest).

Calcium oscillations in the stochastic and deterministic models are shown in Fig. 5A. According to our previous results [8], the average value of 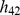 over the cluster of 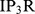 primarily regulates the termination and regeneration of individual spikes. This can be seen in the stochastic model by projecting the solution on the 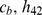 phase plane (Fig. 5B). Upon an initial 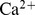 release from one or more 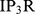, a large spike is generated by Ca^2+^-induced 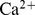 release (via the 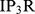) during which time a decreasing 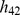 gradually decreases the average open probability of the clustered 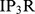. The spike is terminated when 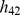 is too small to allow further 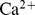 release. This phenomenon is qualitatively reproduced by the deterministic model (Fig. 5D). In both the stochastic and deterministic models the decrease in average 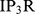 open probability of a cluster of 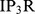 caused by 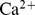 inhibition is the main reason for the termination of each spike.

According to Figs. 5B and D, regeneration of each spike requires a return of 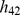 back to a relatively high value (i.e., recovery of the 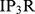 from inhibition by 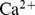). The deterministic model sets a clear threshold for the regeneration, as can be seen in Fig. 5C, where an upstroke in 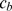 occurs when the trajectory creeps beyond the sharp “knee” of the white curve. When the trajectory reaches the knees of the white curve it is forced to jump across to the other stable branch of the critical manifold, resulting in a fast increase in 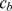 followed by a relatively fast increase in 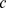 (seen by combining Figs. 5C and D).

In contrast, the stochastic model enlarges the contributions of individual 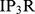 so that the generation of each spike is also effectively driven by random 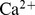 release through the 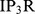, which can be seen in the inset of Fig. 5B where the site of spike initiation (blue bar) exhibits significantly greater variation than that of spike termination (green bar). In spite of this, the essential similarities in phase plane behavior result in both deterministic and stochastic models making the same qualitative predictions in response to perturbations, such as changes in 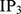 concentration (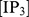), 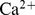 influx or efflux. In the following, we illustrate this by investigating a number of experimentally testable predictions. Due to the extensive importance of frequency encoding in many 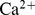-dependent processes, we focus particularly on the change of oscillation frequency in response to parameter perturbations. As a side issue we also investigate how the oscillation baseline depends on physiologically important parameters.

### Dependence of oscillation frequency on 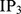 concentration

**Figure 6:**
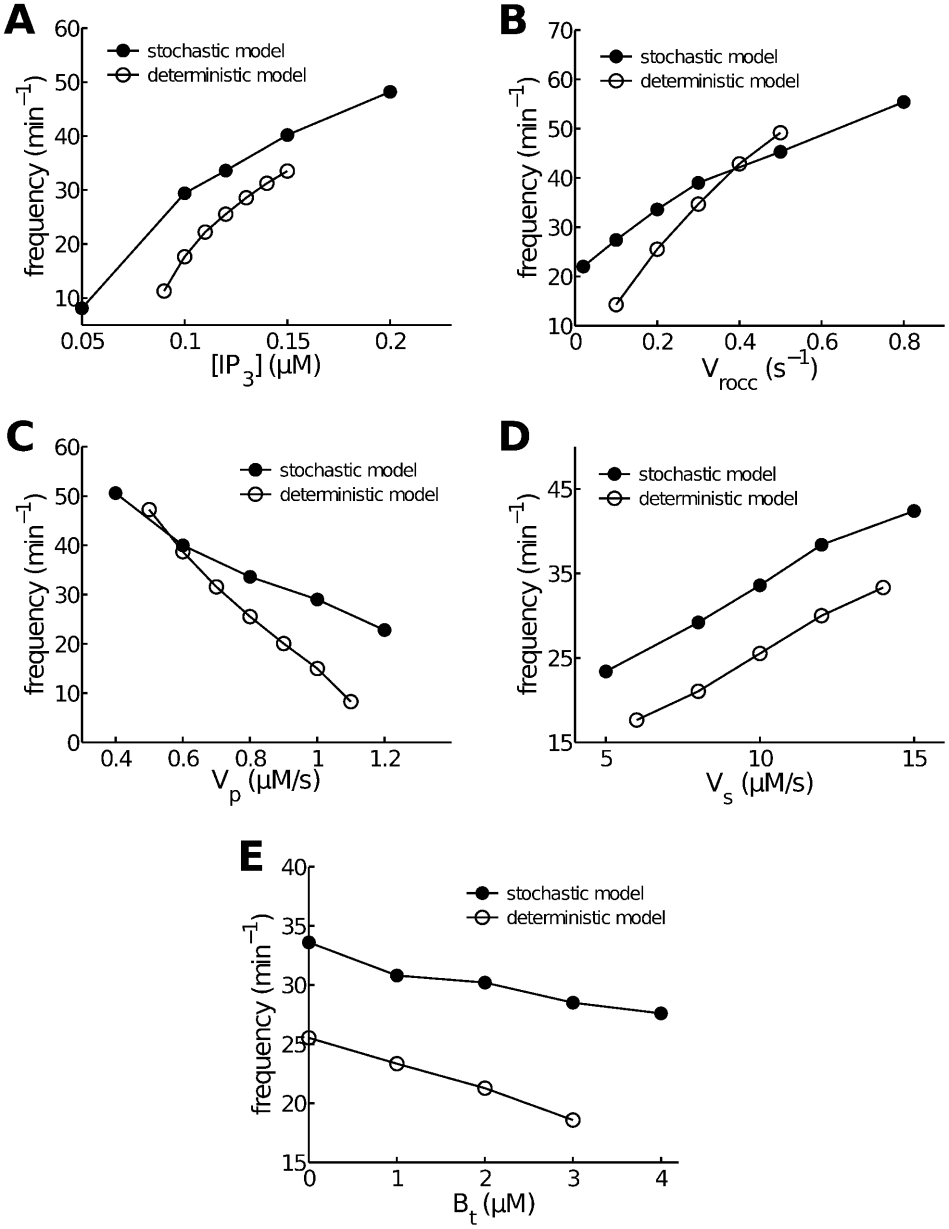
Comparison of parameter-dependent frequency changes in the stochastic and deterministic models. All curves are computed at 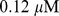 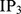 except in panel A, which uses a variety of 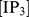. Other parameters are set at their default values given in Table 1. **A**: as 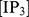 increases, 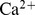 oscillations in both models increase in frequency. **B**: as 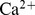 influx increases (modeled by an increase in receptor-operated calcium channel flux coefficient 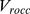), so does the oscillation frequency in both models. **C**: as 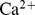 efflux increases (modeled by an increase in plasma pump expression 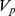), oscillation frequency decreases. **D**: as SERCA pump expression, 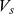, increases, so does oscillation frequency. **E**: as total buffer concentration, 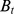, increases, oscillation frequency decreases.

In many cell types a moderate increase in 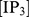 increases the 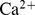 oscillation frequency (see Fig. 2A in [11], Fig. 4E in [16] and Fig. 6B in [17]), a result that is reproduced by both model types (Fig. 6A). As 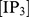 increases, the stochastic model increases the probability of the initial 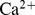 release through the first open 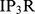 and of the following 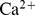 release, thus shortening the average ISI. Although the oscillatory region of the deterministic model is strictly confined by bifurcations which do not apply to the stochastic model, the deterministic model can successfully replicate an increasing frequency by lowering the “knee” of the red curve in Fig. 5D and shortening the time spent from the termination point c to the initiation point a (thus shortening the ISI). Hence, although the deterministic model cannot be used to predict the exact values of 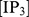 at which the oscillations begin and end, as stochastic effects predominate in these regions, it can be used to predict the correct qualitative trend in oscillation frequency.

### Dependence of oscillation frequency on 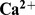 influx and efflux

In many cell types, including ASMC, transmembrane fluxes modulate the total intracellular 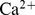 load (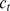) on a slow time scale [16], [18], and thereby modulate the oscillation frequency [19]. Experimental data can be seen in Fig. 8 in [16] and Fig. 2 in [18]. Figs. 6B and C show that both stochastic and deterministic models predict the same qualitative changes in oscillation frequency in response to changes in membrane fluxes (through membrane ATPase pumps and/or 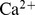 influx channels such as receptor-operated channels or store-operated channels).

**Figure 8:**
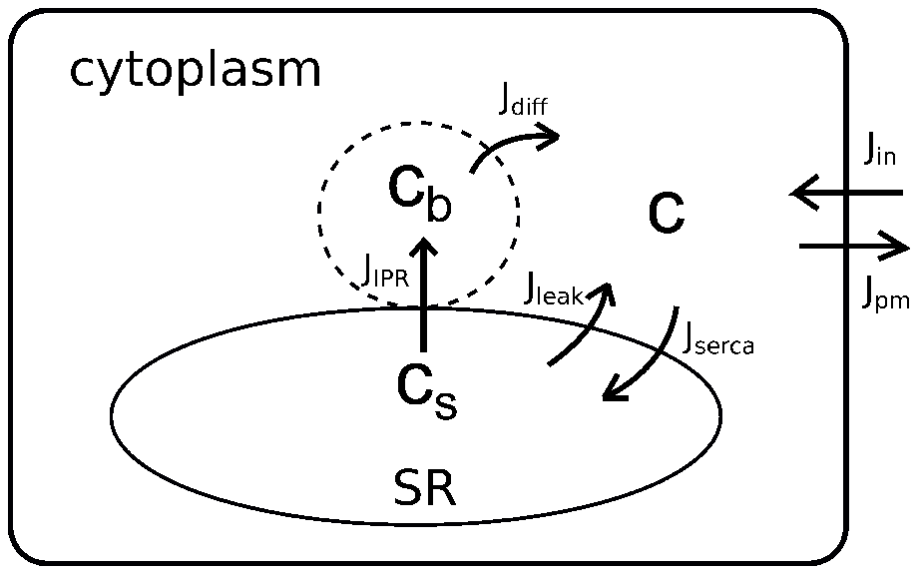
Schematic diagram of the 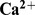 model. 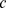
 represents cytoplasmic 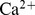 concentration, excluding a small local 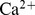 (whose concentration is denoted by 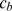) close to the 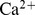 release site (i.e., an 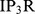 cluster). Upon coordinated openings of the 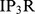, SR 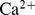 (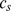) is first released into the local domain (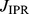) to cause a rapid increase in 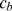. High local 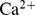 then diffuses to the rest of the cytoplasm (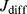), and is eventually pumped back to the SR (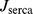).

### Dependence of oscillation frequency on SERCA expression

The level of sarco/endoplasmic reticulum calcium ATPase (SERCA) expression (or capacity) is important for airway remodeling in asthma [20] and ASMC 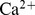 oscillations [21]. We thus investigated the predictions of the two models in response to changes in SERCA expression (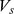). As 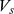 decreases, the deterministic model exhibits a decreasing frequency, in agreement with experimental data (see Figs. 3 and 4 in [21]). The same trend is seen in the stochastic model with only 20 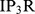 (see Fig. 6D).

### Dependence of oscillation frequency on 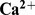 buffer concentration

Calcium buffers have been shown to be able to change the ISI and spike duration, which in turn change the oscillation frequency [15], [22]. We compared the effects on the two models of varying total buffer concentration (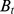) by adding one buffer with relatively fast kinetics to the models (see *Materials and Methods* for details). In both models the frequency decreases as 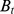 increases (see Fig. 6E), which is consistent with experimental data (Fig. 2B in [18]). This is not surprising, because increasing 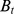 can decrease the effective rates of SR 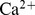 release and reuptake.

### Dependence of oscillation baseline on 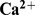 influx and SERCA expression

**Figure 7:**
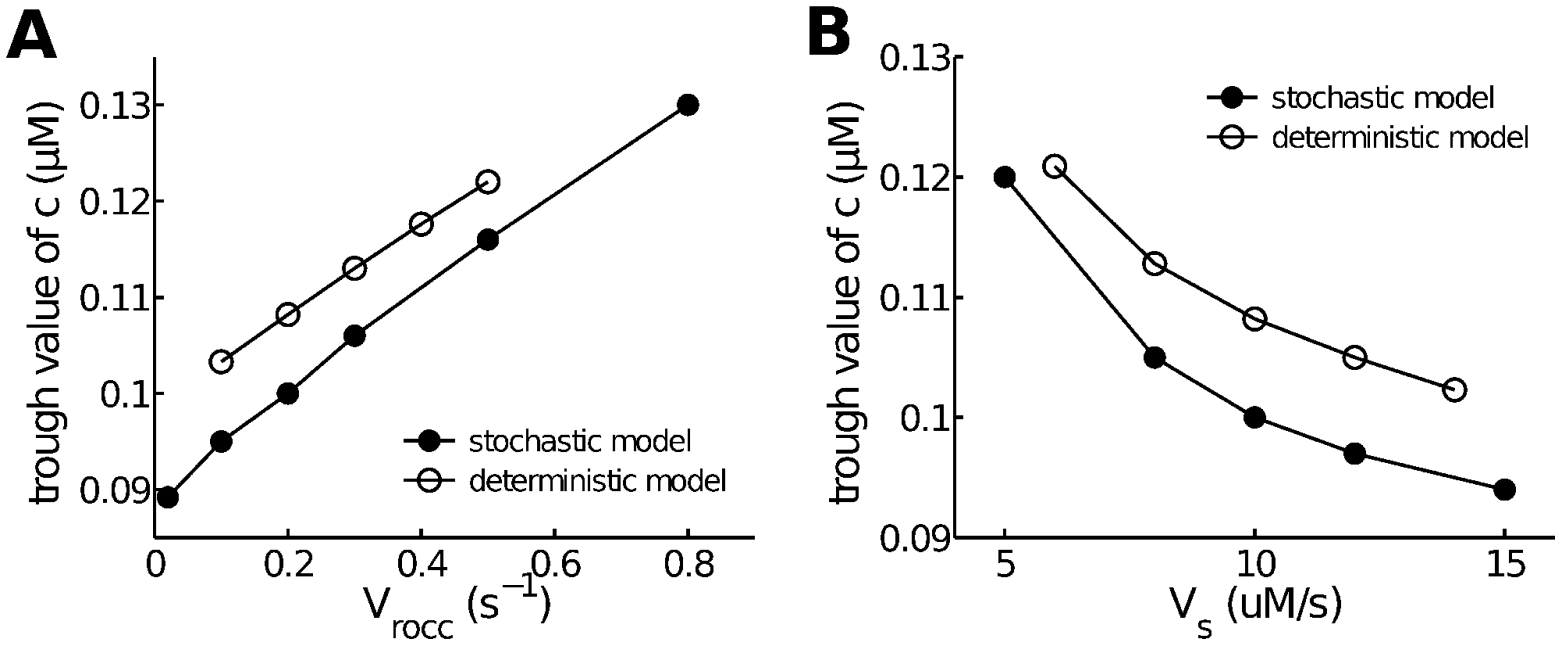
Dependence of calcium oscillation baseline on calcium influx and SERCA expression. **A**: increasing influx (described by 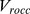) increases the average trough of 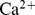 oscillations. **B**: decreasing SERCA expression (described by 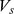) increases the average trough of 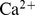 oscillations. All curves are computed at 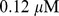 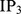.

Sustained elevations of baseline during agonist-induced 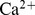 oscillations or transients have been observed experimentally, and are believed to be a result of an increase in 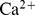 influx caused by opening of membrane 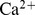 channels [13], [16]. Furthermore, there is evidence showing that decreased SERCA expression could also increase the baseline (Fig. 4 in [21]). Those phenomena are successfully reproduced by both models (see Fig. 7).

## Discussion

In this paper we address two current major questions in the field of 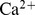 modeling. Firstly, we show that 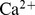 puffs and stochastic oscillations can be reproduced quantitatively by an extremely simple model, consisting only of two states (one open, one closed), with time-dependent transitions between them. This model is obtained by removing the intramodal structure of a more complex model that was determined by fitting a Markov model to single-channel data [7]. We thus show that the internal structure of each mode is irrelevant for function and mode switching is the key mechanism for the control of calcium release. The necessity for time-dependent mode switching is shown not only by the dynamic single-channel data of [4]), but also by the puff data of [9] and our ASMC data.

Secondly, we investigate the role of stochasticity of 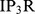 in modeling 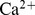 oscillations in ASMC by comparing a stochastic IP_3_R-based 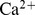 model and its associated deterministic version, for parameters such that both of the models exhibit 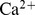 spikes but the stochastic model cannot necessarily be replaced by a mean-field model. We find that a four-variable deterministic model has the same predictive power as the stochastic model, in that it correctly reproduces the process of spike termination and predicts the same qualitative changes in oscillation frequency and baseline in response to a variety of perturbations that are commonly used experimentally. The mechanism for termination of individual spikes is fundamentally a deterministic process controlled by a rapid inhibition induced by the high local 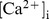 in the 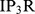 cluster, whereas spike initiation is significantly affected by stochastic opening of 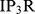. Hence, repetitive 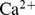 cycling is primarily induced by the time-dependent gating variables governing transitions of the 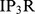 from one mode to another.

Our simplified two-state model of the 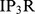 is identical in structure (although not in parameter values) to the well-known model of [23]. It is somewhat ironic that after 20 years of detailed studies of the 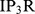 and the construction of a plethora of models of varying complexity, the single-channel data have led us around full circle, back to these original formulations. Excitability is arising via a fast activation followed by a slower inactivation, a combination often seen in physiological processes [24]. Encoding of this fundamental combination results directly from the two-mode structure of the 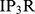. Although similar single-channel data have been used to construct three-mode models [6], [25], neither of these models has yet been used in detailed studies of 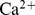 puffs and waves, and it remains unclear whether or not they have a similar underlying structure.

In contrast to previous deterministic ODE models, our four-variable 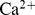 model includes a more accurate 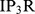 model, as well as local control of clustered 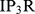 by two distinct 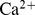 microdomains; one at the mouth of an open 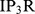, the other inside a cluster of 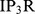. Neglect of either of these microdomains leads to models that either exhibit unphysiological cytoplasmic 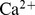 concentrations or fail to reproduce reasonable oscillations. This underlines the importance of taking 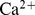 microdomains into consideration when constructing any model. Our microdomain model is highly simplified, with the microdomain being treated simply as a well-mixed compartment. More detailed modeling of spatially-dependent microdomains is possible, and not difficult in principle, but requires far greater computational resources. It is undeniable that a more detailed model, incorporating the full spatial complexity – and possibly stochastic aspects as well – would make, overall, a better predictive tool. However, our goal is to find the simplest models that can be used as predictive tools.

An important similar study is that of Shuai and Jung [26]. They compared the use of Markov and Langevin approaches to the computation of puff amplitude distributions, compared their results with the deterministic limit, and showed that 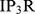 stochasticity does not qualitatively change the type of puff amplitude distribution except for when there are fewer than 10 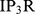. Here, we significantly extend the scope of their study by exploring the effects of 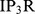 stochasticity on the dynamics of 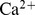 spikes, and we do this in the context of an 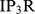 model that has been fitted to single-channel data. Although this is true in a general sense for the Li-Rinzel model, which is based on the DeYoung-Keizer model, which did take into account the opening time distributions of 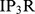 in lipid bilayers, neither model can reproduce the more recent data obtained from on-nuclei patch clamping. When these recent data are taken into account one obtains a model with the same structure, but quite different parameters and behavior.

We find that, in spite of a relatively large variation in spike amplitude which is partially caused by a large variation in ISI (Fig. 5B), the mechanism governing individual spike terminations is the same for both a few or infinitely many 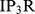, which explains why the one-peak type of amplitude distribution is independent of the choice of 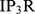 number (see Fig. 6A in [26]).

Another important relevant study was done by Dupont et al. [27], who compared the regularity of stochastic oscillations in hepatocytes for different numbers of 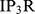 clusters. They found that the impact of 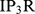 stochasticity on global 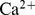 oscillations (in terms of CV) increases as the total cluster number decreases. Our study here extends these results, and demonstrates how well stochastic oscillations can be qualitatively described by a deterministic system, even when there is only a small number of 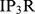 (which appears to be the case for ASMC, in which the wave initiation site is only 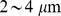 in diameter). Indeed, as we have shown, for the purposes of predictive modeling a simple deterministic model does as well as more complex stochastic simulations.

Ryanodine receptors (RyR) are another important component modulating ASMC 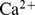 oscillations [16], [28], [29] but are not included in our model. This is because the role of RyR is not fully understood and may be species-dependent; for example, in mouse or human ASMC, RyR play very little role in 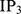-induced continuing 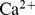 oscillations [17], [30], but this appears not to be true for pigs [28]. Our study focuses on the calcium oscillations in mouse and human (as we did in our experiments) where inclusion of a deterministic model of RyR should have little effect. An understanding of the role of RyR stochasticity and how the 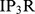 and the RyR interact needs a reliable RyR Markov model, exclusive to ASMC, which is not currently available. Multiple Markov models of the RyR have been developed for use in cardiac cells [31], but these are based on single-channel data from lipid bilayers, and are adapted for the specific context of cardiac cells. Their applicability to ASMC remains unclear.

Although we have not shown that the deterministic model for ASMC has the same predictive power as the stochastic model in all possible cases (which would hardly be possible in the absence of an analytical proof) the underlying similarity in phase plane structure indicates that such similarity is plausible at least. Certainly, we have not found any counterexample to this claim. However, whether or not this claim is true for all cell types is unclear. Some cell types exhibit both local 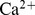 puffs and global 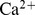 spikes (usually propagating throughout the cells in the form of traveling waves), showing that initiation of such 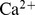 spikes requires a synchronization of 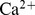 release from more than one cluster of 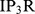 [14]. This type of spiking relies on the hierarchical organization of 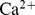 signal pathways, in particular the stochastic recruitment of both individual 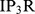 and puffs at different levels [32], and therefore cannot be simply reproduced by deterministic models containing only a few ODEs. However, 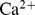 oscillations in ASMC, as observed in lung slices, may not be of this type, as IP_3_R-dependent puffs have not been seen in these ASMC. It thus appears that, in ASMC in lung slices, every 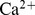 “puff” initiates a wave, resulting in periodic waves with ISI that are governed by the dynamics of individual puffs.

## Materials and Methods

### Ethics Statement

Animal experimentations carried out were approved by the Animal Care and Use Committee of the University of Massachusetts Medical School under approval number A-836-12.

### Lung slice preparation

BALB/c mice (7–10 weeks old, Charles River Breeding Labs, Needham, MA) were euthanized via intraperitoneal injection of 0.3 ml sodium pentabarbitone (Oak Pharmaceuticals, Lake Forest, IL). After removal of the chest wall, lungs were inflated with 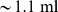 of 1.8% warm agarose in sHBSS via an intratracheal catheter. Subsequently, air (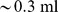) was injected to push the agarose within the airways into the alveoli. The agarose was polymerized by cooling to 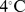. A vibratome (VF-300, Precisionary Instruments, San Jose, CA) was used to make 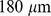 thick slices which were maintained in Dulbecco's Modified Eagle's Media (DMEM, Invitrogen, Carlsbad, CA) at 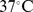 in 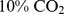/air. All experiments were conducted at 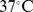 in a custom-made temperature-controlled Plexiglas chamber as described in [17].

### Measurement of 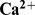 oscillations

Lung slices were incubated in sHBSS containing 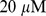 Oregon Green 488 BAPTA-1-AM (Invitrogen), a Ca^2+^-indicator dye, 0.1% Pluronic F-127 (Invitrogen) and 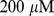 sulfobromophthalein (Sigma Aldrich, St Louis, MO) in the dark at 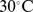 for 1 hour. Subsequently, the slices were incubated in 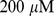 sulfobromophthalein for 30 minutes. Slices were mounted on a cover-glass and held down with 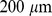 mesh. A smaller cover-glass was placed on top of the mesh and sealed at the sides with silicone grease to facilitate solution exchange. Slices were examined with a custom-built 2-photon scanning laser microscope with a 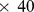 oil immersion objective lens and images recorded at 30 images per second using Videosavant 4.0 software (IO Industries, Montreal, Canada). Changes in fluorescence intensity (which represent changes in 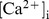) were analyzed in an ASMC of interest by averaging the grey value of a 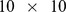 pixel region using custom written software. Relative fluorescence intensity (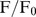) was expressed as a ratio of the fluorescence intensity at a particular time (F) normalized to the initial fluorescence intensity (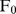).

### The calcium model

Inhomogeneity of cytoplasmic 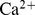 concentration not only exists around individual channel pores of the 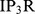, where a nearly instantaneous high 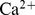 concentration at the pore (denoted by 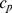) leads to a very sharp concentration profile, but is also seen inside an 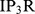 cluster where the average cluster 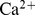 concentration (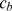) is apparently higher than that of the surrounding cytoplasm (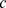) [33]. This indicates that during 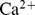 oscillations each 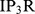 is controlled by either the pore 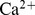 concentration (when it is open) or the cluster 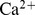 concentration (when it is closed). Neither of these local concentrations influence cell membrane fluxes or the majority of SERCAs, which we assume to be distributed outside the cluster.

The scale separation between the pore 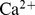 concentration and the cluster 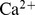 concentration allows to treat 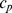 as a parameter, providing a simpler way of modeling local 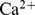 events (like 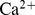 puffs) that has been used in several previous studies [8], [34], [35]. However, evolution of the cluster concentration and wide-field cytoplasm 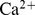 concentration are not always separable, so an additional differential equation for the cluster 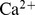 is necessary.

A schematic diagram of the model is shown in Fig. 8. The corresponding ODEs are 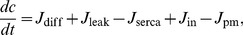
(1)

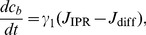
(2)

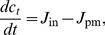
(3)where 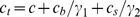 representing total intracellular 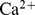 concentration, and thus SR 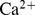 concentration, 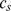 is given by 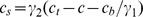. 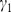 and 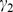 are the volume ratios given in Table 1. 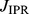 is the flux through the 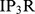, 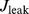 is a background 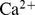 leak out of the SR, and 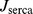 is the uptake of 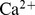 into the SR by SERCA pumps. 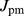 is the flux through plasma pump, and 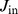 represents a sum of main 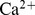 influxes including 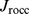 (receptor-operated 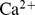 channel), 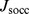 (store-operated 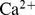 channel) and 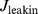 (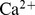 leak into the cell). 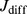 coarsely models the diffusion flux from cluster microdomain to the cytoplasm. Details of the fluxes are

**Table 1:** Parameter values of the stochastic calcium model.

Parameter	Description	Value/Units
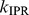	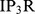 flux coefficient	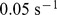
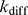	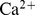 diffusional flux coefficient	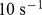
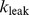	SR leak flux coefficient	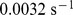
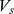	maximum capacity of SERCA	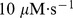
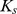	SERCA half-maximal activating 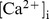	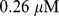
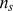	Hill coefficient for SERCA	1.75
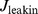	plasma membrane leak influx	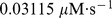
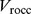	ROCC flux coefficient	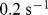
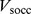	maximum capacity of SOCC	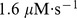
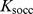	SOCC dissociation constant	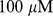
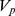	maximum capacity of plasma pump	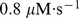
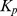	half-maximal activating 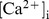 of plasma pump	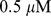
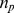	Hill coefficient for plasma pump	2
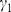	the cytoplasmic-to-microdomain volume ratio	100
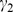	the cytoplasmic-to-SR volume ratio	10
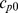	an instantaneous high 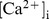 at open channel pore when 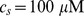	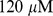
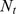	total number of 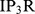 channels	20

• Different formulations of 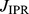 give different types of models:
  a) For the stochastic model, 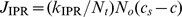 where 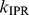 is the maximum conductance of a cluster of 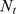 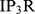 (here 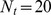). 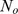 is the number of open 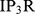 determined by the states of 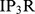.
  b) For the deterministic model we set 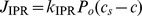 where 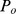 is the 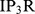 open probability, a continuous analogue of 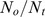.
  To calculate 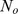 and 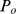, we use the 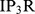 model of [7], [8], with minor modifications described later.

• 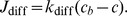

• 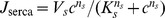 where 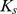 and 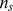 are obtained from [36].

• 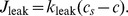

• 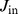 includes a basal leak (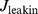), receptor-operated calcium channel (ROCC, 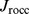), store-operated calcium channel (SOCC, 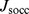). By using the 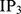 concentration (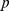) as a surrogate indicator of MCh concentration, we assume that 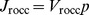. SOCC is modeled by 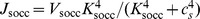 [13].

• 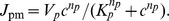

Calcium concentration at open channel pore (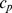) does not explicitly appear in the equations but is used in the 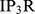 model introduced later. 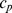 is assumed to be proportional to SR 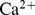 concentration (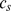) and is therefore simply modeled by 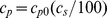 where 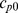 is the value corresponding to 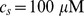. Alternatively, 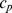 can also be assumed to be a large constant (say greater than 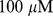) without fundamentally altering the model dynamics. The choice of 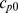 is not critical as long as it is sufficiently large to play a role in inactivating the open channels. All the parameter values are given in Table 1.

### The data-driven 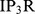 model

The 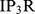 model used in our ASMC calcium model is an improved version of the Siekmann 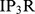 model which is a 6-state Markov model derived by fitting to the stationary single channel data using Markov chain Monte Carlo (MCMC) [5], [7], [8]. Fig. 1 has shown the structure of the 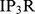 model which is comprised of two modes; the drive mode, containing three closed states 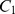, 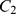, 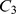 and one open state 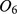, and the park mode, containing one closed state 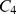 and one open state 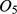. The transition rates in each mode are constants (shown in Table 2), but 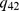 and 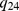 which connect the two modes are 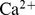-/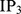-dependent and are formulated as 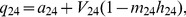
(4)

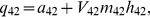
(5)where 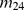, 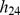, 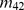 and 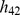 are 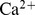-/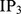-modulated gating variables. 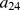, 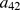, 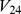 and 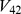 are either functions of 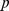 or constants and are given later. We assume the gating variables obey the following differential equation, 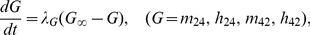
(6)where 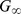 is the equilibrium and 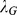 is the rate at which the equilibrium is approached. Those equilibria are functions of 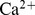 concentration at the cytoplasmic side of the 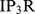, denoted by 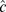 in the equations, equal to either 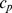 or 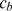 depending on the state of the channel). They are assumed to be 
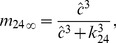
(7)

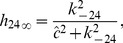
(8)

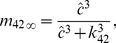
(9)

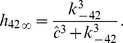
(10)


**Table 2:** Parameter values of the 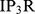 model.

Parameter	Value/Units	Parameter	Value/Units
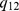	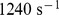	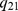	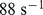
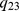	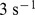	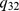	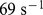
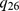	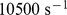	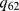	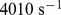
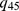	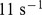	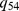	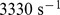
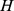	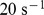	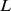	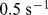
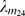	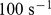	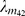	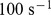
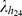	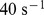		

Hence, we have stationary expressions of 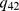 and 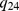, 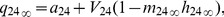
(11)

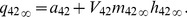
(12)


**Figure 9:**
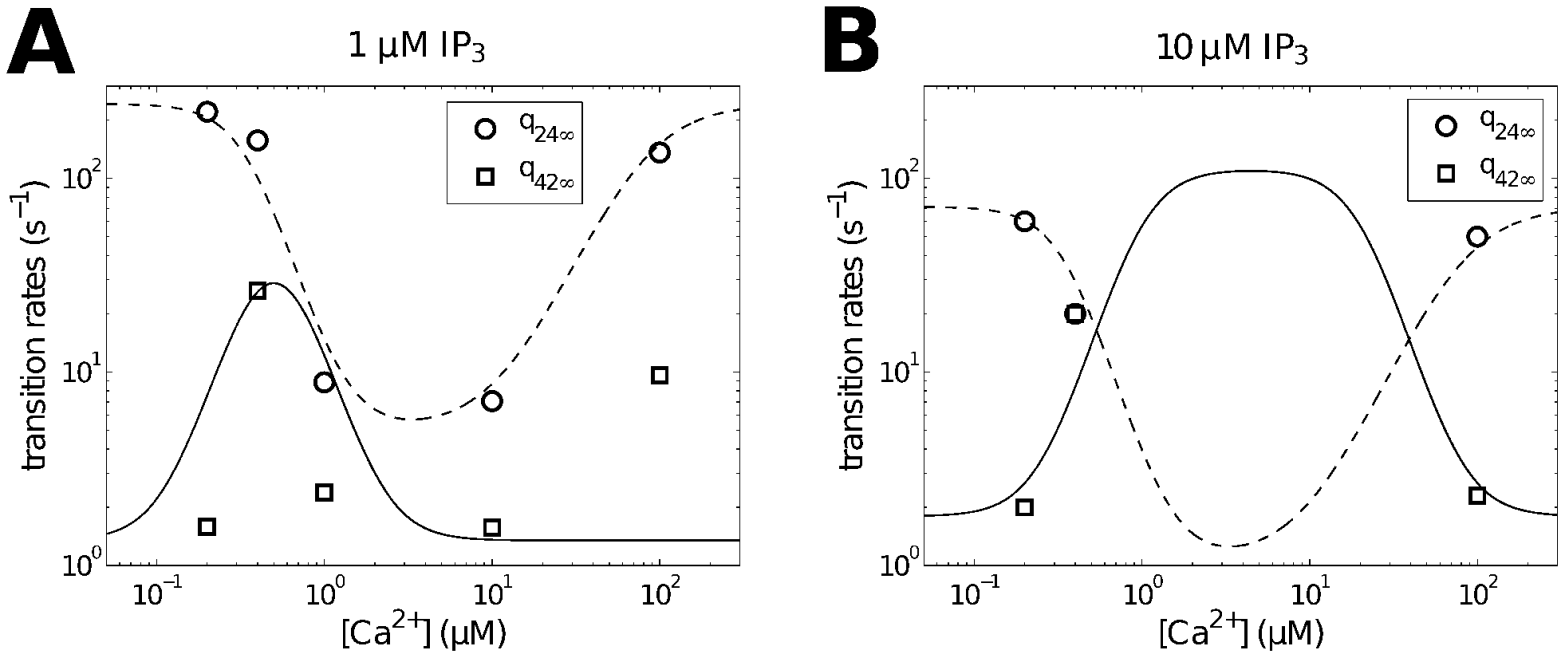
Stationary data and fits of 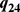 and 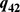. Stationary transition rates of 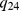 and 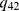, 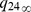 and 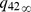, as functions of 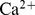 concentration were estimated and fitted for two 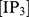, 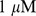 (**A**) and 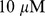 (**B**). Circles and squares represent the means of 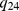 and 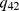 distributions computed by MCMC simulation [7]. Note that MCMC failed to determine the values of 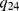 and 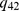 at 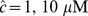 for 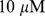 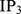, as the 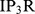 was almost in the drive mode for these cases. The corresponding fitting curves (solid for 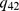; dashed for 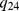) are produced using Eqs. 7–12.

**Figure 10:**
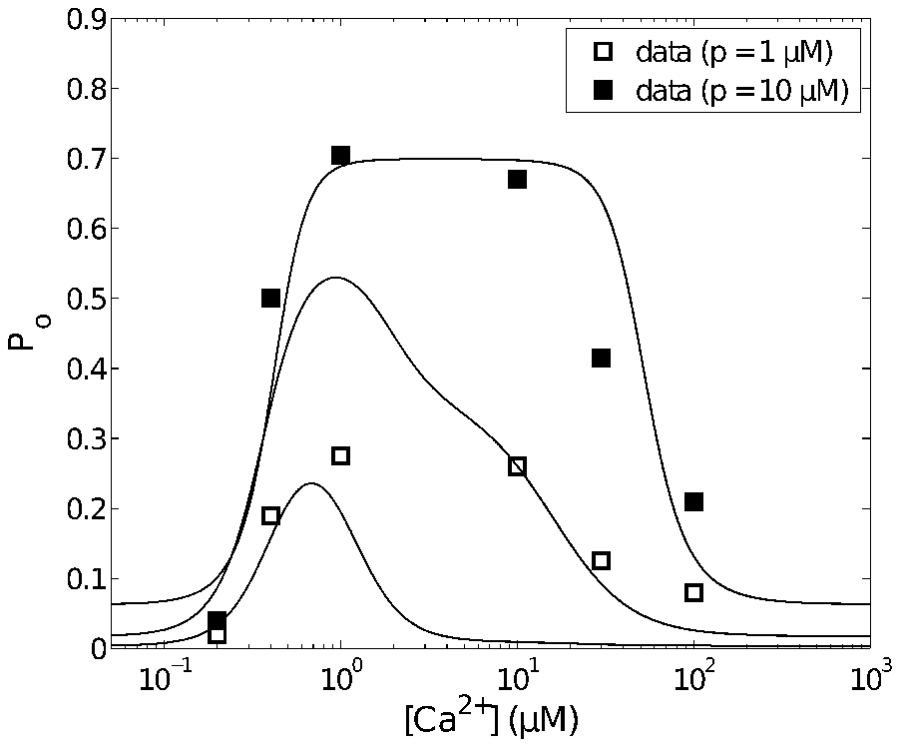
Open probability curves for various 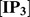. 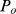
 is equal to the sum of probabilities of the 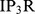 in 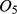 and 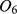. Three representative curves correspond to 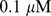, 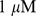 and 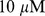 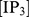 (from bottom to top) respectively. Data (average open probability) are from [5].

The expressions of 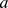
s, 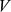
s, 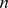
s and 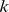
s are chosen as follows so that Eq. 11 and Eq. 12 capture the correct trends of experimental values of 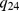 and 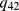 (see Fig. 9) and generate relatively smooth open probability curves (see Fig. 10), 
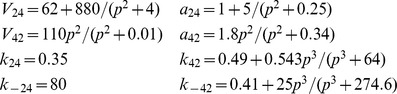



Note that the above formulas are different from the relatively complicated formulas used in [8]. The rates, 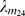, 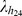 and 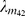, are constants estimated by using dynamic single channel data [4] and given in Table 2, whereas 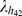 is not clearly revealed by experimental data. However we have shown that it should be relatively large for high 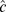 but relatively small for low 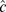 for reproducing experimental puff data [8]. By introducing two 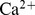 concentrations, 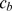 and 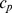, 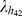 and the state of the 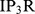 channel become highly correlated, so that we can assume 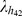 is a relatively large value 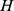 if the channel is open and is a relatively small value 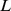 if the channel is closed. Hence, 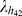 is modeled by the logic function 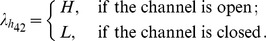



Values of 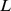 and 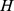 are chosen so that simulated 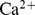 oscillations in ASMC are comparable to experimental observations.

### The 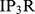 model reduction

Here we reduce the 6-state model to a 2-state open/closed model. The reduction takes the following steps:

- The sum of the probabilities of 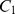, 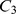 and 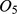 is less than 0.03 for any 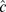, so they are either rarely visited by the 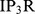 or have a very short dwell time. This implies they have very little contribution to the 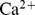 dynamics. Therefore, we completely remove the three states from the full model.
- Transition rates of 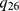 and 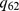 are about 2 orders larger than that of 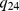 and 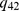, which allows us to omit the fast transitions by taking a quasi-steady state approximation. This change will affect two aspects. First, we have 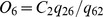 which allows us to combine 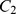 and 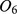 to be a new state 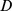, which satisfies 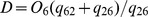. Although this means 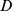 is a partially open state with an open probability of 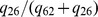, it can be used as an fully open state in the stochastic simulations by multiplying the maximum 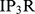 flux conductance 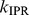 by a factor of 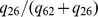. Secondly, 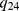 needs to be rescaled by 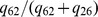, i.e., the effective closing rate is 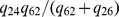.
- Due to the combination of 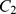 and 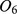, 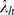 is accordingly modified to

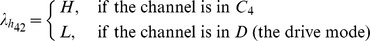

Hence, the reduced two-state model contains one “open” state 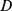 and one closed state 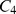 with the opening transition rate of 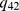 and the closing transition rate of 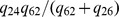.

### Deterministic formulation of the stochastic model

Based on the stochastic calcium model and the reduced 2-state 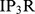 model, we construct a deterministic model. We need to modify three things that are used in the stochastic model but inapplicable to fast simulations of the deterministic model. The first is the discrete number of open channels; the second is state-dependent use of 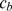 and 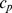 in calculating 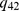 and 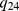; the last is the logic expression of 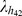. Details of the modifications are as follows,

- The fraction of open channels (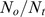) is replaced by open probability 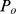 which is 70% of the probability of state 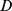.
- In the stochastic simulations, 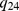 which only controls the 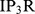 closing is primarily governed by 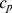, whereas 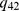 which controls 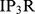 opening is mainly governed by 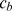. Therefore, in the deterministic model, we separate the functions of 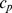 and 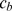 by assuming 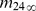 and 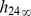 are functions of 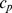 only whereas 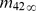 and 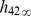 are functions of 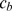 only. That is, 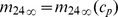, 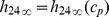, 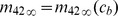 and 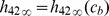. Here 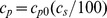 as defined before.
- To describe an average rate that infinitely many receptors are rapidly inhibited by high 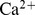 concentration but slowly restored from 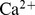-inhibition. 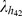 is proposed to be

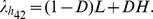

Based on the above changes, the full deterministic model containing 8 ODEs is presented as follows,
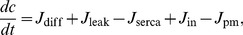
(13)

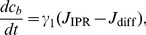
(14)

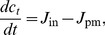
(15)

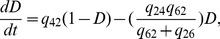
(16)

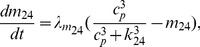
(17)

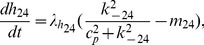
(18)

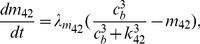
(19)

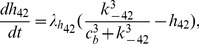
(20)where 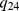 and 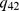 are functions of the gating variables given by Eqs. 4 and 5. All the fluxes are the same as those of the stochastic model except 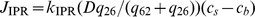. All the parameter values of the deterministic model are the same as those of the stochastic model and are therefore given in Tables 1 and 2.

### Reduction of the full deterministic model

The full deterministic model contains 8 variables which make the model difficult to implement and analyze. Thus, we reduce the full model to a minimal model that still captures the crucial features of the full model. First of all, 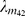, 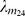 and 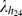 are sufficiently large so that we can assume they instantaneously follow their equilibrium functions. Therefore, by taking quasi-steady state approximation to 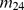, 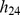 and 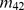, we remove the three time-dependent variables from the full model.

By now, the full model has been reduced to a 5D model, 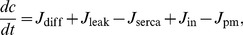
(21)

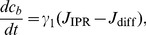
(22)

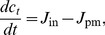
(23)

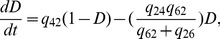
(24)

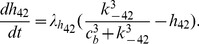
(25)


Second, the rate of change of 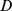 approaching its equilibrium, 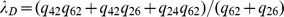 (calculated from Eq. 24), is at least one order larger than those of 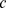, 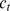 and 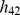, indicating that taking the quasi-steady state approximation to Eq. 24 could not significantly affect the evolutions of 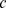, 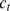 and 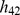. That is, 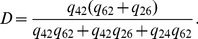
(26)


We emphasize here that the theory of the quasi-steady state approximation has not yet been well established, particularly about the rigorous conditions under which such a reduction is valid. Thus, our criterion of judging the validity of the reduction is checking whether the solutions of the reduced model are capable of qualitatively reproducing that of its original model. For this model, we find the reduction works. Hence, the full model is eventually reduced to a 4D model summarized as follows, 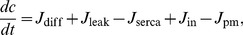
(27)

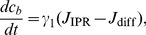
(28)

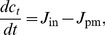
(29)

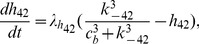
(30)where 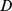 is given by Eq. 26.

### Inclusion of calcium buffers

To check the effect of calcium buffers on oscillation frequency, we introduce a stationary buffer (no buffer diffusion), as mobile buffers are too complicated to be included in the current deterministic model. Since we have two different cytoplasmic 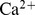 concentrations, 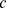 and 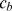, two pools of buffer with the same kinetics should be considered. Hence, the inclusion of a stationary calcium buffer is modeled by the following system, 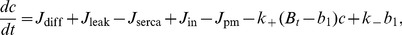
(31)

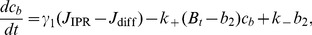
(32)

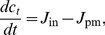
(33)

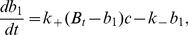
(34)

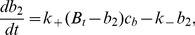
(35)where 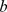 (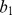 and 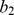) and 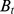 represent the concentrations of 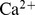-bound buffer and total buffer respectively. 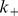 and 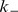 are the rates of 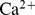-binding and 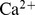-dissociation, indicating how fast the time scale of the buffer dynamics is. Fast buffer refers to the buffer with relatively large 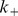. In the simulations, we use a fast buffer with 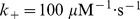 and 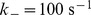 and vary 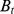 to test if the stochastic model and the deterministic model have a qualitatively similar 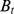-dependency. Results are given in Fig. 6E.

### Numerical methods and tools for deterministic and stochastic simulations

For the stochastic model, Eqs. 1–3 and ODEs of the four gating variables in the 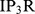 model are solved by the fourth-order Runge-Kutta method (RK4) and the stochastic states of 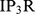 determined by the 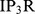 model are solved by using a hybrid Gillespie method with adaptive timing [37]. The maximum time step size is set to be either 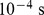 (for the 6-state 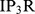 model) or 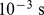 (for the reduced 2-state 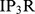 model). All the computations are done with MATLAB (The MathWorks, Natick, MA) and the codes are provided in Supporting information (Text S1–S2). For the deterministic model, we use ode15s, an ODE solver in MATLAB. Accuracy is controlled by setting an absolute tolerance of 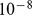 applied to all the variables.

### Statistical analysis

Data analysis is performed on the 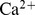 traces with relatively stable baselines and less noise. A moving average of every 3 data points is used to improve the data by smoothing out short-term fluctuations (Fig. 2A is an improved result). Due to large variations in baseline, amplitude, and level of noise in data, we used two thresholds to get samples: a low threshold, 20% of the amplitude of the largest spike above the baseline, to initially filter baseline noise out; and a relatively high threshold, 50% of the amplitude of the largest spike above the baseline, to further remove small spikes that cannot initiate waves. For simulated stochastic traces of variable 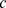, we first convert it to fluorescence ratio (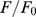) by using 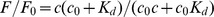 where the dissociation constant of Oregon Green 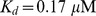 and resting 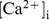 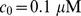. We then used the same sampling procedure mentioned above to obtain samples. After samples are chosen, ISIs and spike durations are calculated based on the low threshold. Simulated traces used to calculate average frequency are about 200–400 seconds long. All the samplings and linear least-squares fittings are implemented using MATLAB (see Text S3–S4 for Matlab codes).

## Supporting Information

Dataset S1
**ASMC calcium fluorescence trace data.** The data files are in Excel format and compressed in a zip file. Each Excel file has a name showing their information. For example, “S2_SMC6_MCh200nM” means data are from ASMC No. 6 in lung slice No. 2 by using 200 nM MCh. In each file, there are four columns which represent (from left to right) time(s), fluorescence intensity, 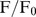 and average 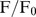.(ZIP)

Text S1
**Matlab code for simulation using 6 state** 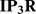 **model.**
(DOCX)

Text S2
**Matlab code for simulation using 2 state** 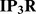 **model.**
(DOCX)

Text S3 **Matlab code for experimental data analysis.**
(DOCX)

Text S4 **Matlab code for simulation analysis.**
(DOCX)

## References

[1] De Young GW, Keizer J (1992) A single-pool inositol 1, 4, 5-trisphosphate-receptor-based model for agonist-stimulated oscillations in Ca^2+^ concentration. Proc Natl Acad Sci USA 89: 9895–9899. 1329108 10.1073/pnas.89.20.9895 PMC50240

[2] Dupont G, Goldbeter A (1993) One-pool model for Ca^2+^ oscillations involving Ca^2+^ and inositol 1,4,5-trisphosphate as co-agonists for Ca^2+^ release. Cell Calcium 14: 311–322. 8370067 10.1016/0143-4160(93)90052-8

[3] Atri A, Amundson J, Clapham D, Sneyd J (1993) A single-pool model for intracellular calcium oscillations and waves in the *Xenopus laevis* oocyte. Biophys J 65: 1727–1739. 8274661 10.1016/S0006-3495(93)81191-3 PMC1225900

[4] Mak DOD, Pearson JE, Loong KPC, Datta S, Fernández-Mongil M, et al (2007) Rapid ligand-regulated gating kinetics of single IP3R Ca^2+^ release channels. EMBO Rep 8: 1044–1051. 17932510 10.1038/sj.embor.7401087 PMC2247393

[5] Wagner LE, Yule DI (2012) Differential regulation of the InsP3 receptor type-1 and -2 single channel properties by InsP3, Ca^2+^ and atp. J Physiol 590: 3245–3259. 22547632 10.1113/jphysiol.2012.228320 PMC3459040

[6] Ullah G, Mak DOD, Pearson JE (2012) A data-driven model of a modal gated ion channel: the inositol 1,4,5-trisphosphate receptor in insect sf9 cells. J Gen Physiol 140: 159–173. 22851676 10.1085/jgp.201110753 PMC3409100

[7] Siekmann I, Wagner LE, Yule DI, Crampin EJ, Sneyd J (2012) A kinetic model for IP3R type i and type ii accounting for mode changes. Biophys J 103: 658–668. 22947927 10.1016/j.bpj.2012.07.016 PMC3443778

[8] Cao P, Donovan G, Falcke M, Sneyd J (2013) A stochastic model of calcium puffs based on single-channel data. Biophys J 105: 1133–1142. 24010656 10.1016/j.bpj.2013.07.034 PMC3852038

[9] Smith IF, Parker I (2009) Imaging the quantal substructure of single IP3R channel activity during Ca^2+^ puffs in intact mammalian cells. Proc Natl Acad Sci USA 106: 6404–6409. 19332787 10.1073/pnas.0810799106 PMC2669345

[10] Brumen M, Fajmut A, Dobovišek A, Roux E (2005) Mathematical modelling of Ca^2+^ oscillations in airway smooth muscle cells. J Biol Phys 31: 515–524. 23345915 10.1007/s10867-005-2409-4 PMC3456323

[11] Sneyd J, Tsaneva-Atanasova K, Reznikov V, Bai Y, Sanderson MJ, et al (2006) A method for determining the dependence of calcium oscillations on inositol trisphosphate oscillations. Proc Natl Acad Sci USA 103: 1675–1680. 16446452 10.1073/pnas.0506135103 PMC1413622

[12] Wang IY, Bai Y, Sanderson MJ, Sneyd J (2010) A mathematical analysis of agonist- and kcl-induced Ca^2+^ oscillations in mouse airway smooth muscle cells. Biophys J 98: 1170–1181. 20371316 10.1016/j.bpj.2009.12.4273 PMC2849087

[13] Croisier H, Tan X, Perez-Zoghbi JF, Sandrson MJ, Sneyd J, et al (2013) Activation of store-operated calcium entry in airway smooth muscle cells: insight from a mathematical model. PLoS ONE 8(7): e69598 10.1371/journal.pone.0069598 23936056 PMC3723852

[14] Marchant JS, Parker I (2001) Role of elementary Ca^2+^ puffs in generating repetitive Ca^2+^ oscillations. EMBO J 20: 65–76. 11226156 10.1093/emboj/20.1.65 PMC140189

[15] Skupin A, Kettenmann H, Winkler U, Wartenberg M, Sauer H, et al (2008) How does intracellular Ca^2+^ oscillate: by chance or by the clock? Biophys J 94: 2404–2411. 18065468 10.1529/biophysj.107.119495 PMC2257893

[16] Perez JF, Sanderson MJ (2005) The frequency of calcium oscillations induced by 5-ht, ach, and kcl determine the contraction of smooth muscle cells of intrapulmonary bronchioles. J Gen Physiol 125: 535–553. 15928401 10.1085/jgp.200409216 PMC2234076

[17] Bai Y, Edelmann M, Sanderson MJ (2009) The contribution of inositol 1,4,5-trisphosphate and ryanodine receptors to agonist-induced Ca^2+^ signaling of airway smooth muscle cells. Am J Physiol Lung Cell Mol Physiol 297: L347–L361. 19465516 10.1152/ajplung.90559.2008 PMC2742787

[18] Bird GSJ, Putney JW (2005) Capacitative calcium entry supports calcium oscillations in human embryonic kidney cells. J Physiol 562(3): 697–706. 15513935 10.1113/jphysiol.2004.077289 PMC1665541

[19] Sneyd J, Tsaneva-Atanasova K, Yule DI, Thompson JL, Shuttleworth TJ (2004) Control of calcium oscillations by membrane fluxes. Proc Natl Acad Sci USA 101: 1392–1396. 14734814 10.1073/pnas.0303472101 PMC337063

[20] Mahn K, Hirst SJ, Ying S, Holt MR, Lavender P, et al (2009) Diminished sarco/endoplasmic reticulum Ca^2+^ atpase (serca) expression contributes to airway remodelling in bronchial asthma. Proc Natl Acad Sci USA 106: 10775–10780. 19541629 10.1073/pnas.0902295106 PMC2699374

[21] Sathish V, Leblebici F, Kip SN, Thompson MA, Pabelick CM, et al (2008) Regulation of sarcoplasmic reticulum Ca^2+^ reuptake in porcine airway smooth muscle. Am J Physiol Lung Cell Mol Physiol 294: L787–L796. 18245264 10.1152/ajplung.00461.2007

[22] Zeller S, Rüdiger S, Engel H, Sneyd J, Warnecke G, et al (2009) Modeling of the modulation by buffers of Ca^2+^ release through clusters of IP_3_ receptors. Biophys J 97: 992–1002. 19686646 10.1016/j.bpj.2009.05.050 PMC2726323

[23] Li Y, Rinzel J (1994) Equations for InsP_3_ receptor-mediated [Ca^2+^]_i_ oscillations derived from a detailed kinetic model: a hodgkin-huxley like formalism. J Theor Biol 166: 461–473. 8176949 10.1006/jtbi.1994.1041

[24] Keener J, Sneyd J (2009) Mathematical Physiology, Second Edition. Springer, New York.

[25] Ionescu L, White C, Cheung KH, Shuai J, Parker I, et al (2007) Mode switching is the major mechanism of ligand regulation of InsP_3_ receptor calcium release channels. J Gen Physiol 130: 631–645. 17998395 10.1085/jgp.200709859 PMC2151663

[26] Shuai JW, Jung P (2002) Stochastic properties of Ca^2+^ release of inositol 1,4,5-trisphosphate receptor clusters. Biophys J 83: 87–97. 12080102 10.1016/S0006-3495(02)75151-5 PMC1302129

[27] Dupont G, Abou-Lovergne A, Combettes L (2008) Stochastic aspects of oscillatory Ca^2+^ dynamics in hepatocytes. Biophys J 95: 2193–2202. 18515398 10.1529/biophysj.108.133777 PMC2517042

[28] Kannan MS, Prakash YS, Brenner T, Mickelson JR, Sieck GC (1997) Role of ryanodine receptor channels in Ca^2+^ oscillations of porcine tracheal smooth muscle. Am J Physiol 272: L659–L664. 9142939 10.1152/ajplung.1997.272.4.L659

[29] Tazzeo T, Zhang Y, Keshavjee S, Janssen LJ (2008) Ryanodine receptors decant internal Ca^2+^ store in human and bovine airway smooth muscle. Eur Respir J 32: 275–284. 18353852 10.1183/09031936.00167007

[30] Ressmeyer AR, Bai Y, Delmotte P, Uy KF, Thistlethwate P, et al (2010) Human airway contraction and formoterol-induced relaxation is determined by Ca^2+^ oscillations and Ca^2+^ sensitivity. Am J Respir Cell Mol Biol 43: 179–191. 19767449 10.1165/rcmb.2009-0222OC PMC2937231

[31] Soeller C, Cannell MB (2004) Analysing cardiac excitation-contraction coupling with mathematical models of local control. Prog Biophys Mol Biol 85: 141–162. 15142741 10.1016/j.pbiomolbio.2003.12.006

[32] Thurley K, Skupin A, Thul R, Falcke M (2012) Fundamental properties of Ca^2+^ signals. Biochim Biophys Acta 1820(8): 1185–1194. 22040723 10.1016/j.bbagen.2011.10.007

[33] Dickinson G, Parker I (2013) Factors determining the recruitment of inositol trisphosphate receptor channels during calcium puffs. Biophys J 105: 2474–2484. 24314078 10.1016/j.bpj.2013.10.028 PMC3853323

[34] Rüdiger S, Shuai JW, Sokolov IM (2010) Law of mass action, detailed balance, and the modeling of calcium puffs. Phys Rev Lett 105(4): 048103 10.1103/PhysRevLett.105.048103 20867887

[35] Rüdiger S, Jung P, Shuai J (2012) Termination of Ca^2+^ release for clustered IP_3_R channels. PLoS Comput Biol 8(5): e1002485 10.1371/journal.pcbi.1002485 22693433 PMC3364945

[36] Chandrasekera PC, Kargacim ME, Deans JP, Lytton J (2009) Determination of apparent calcium affinity for endogenously expressed human sarco(endo)plasmic reticulum calcium-atpase isoform serca3. Am J Physiol Cell Physiol 296: C1105–C1114. 19225163 10.1152/ajpcell.00650.2008

[37] Rüdiger S, Shuai JW, Huisinga W, Nagaiah C, Warnecke G, et al (2007) Hybrid stochastic and deterministic simulations of calcium blips. Biophys J 93: 1847–1857. 17496042 10.1529/biophysj.106.099879 PMC1959544

