# Supplementary material for: A Deterministic Model Predicts the Properties of Stochastic Calcium Oscillations in Airway Smooth Muscle Cells
Source: PLoS Comput Biol. 2014 Aug 14;10(8):e1003783. doi: 10.1371/journal.pcbi.1003783 (PMC4133161; doi:10.1371/journal.pcbi.1003783)
Supplement: Text S2 — Matlab code for simulation using 2 state model. (DOCX) [file pcbi.1003783.s003.docx]

%%

clear all

format long

clc

% construct vectors and set initial conditions

time=[0]; % time

c0=0.1; % resting calcium concentration

cb=c0; % initial microdomian calcium concentration

c=c0; % initial cytosolic calcium concentration

ct0=48.5; % total calcium concentration

ct=ct0;

gamma1=100;gamma2=10;

Num_IPR=20; % number of IP3Rs

state=4*ones(Num_IPR,1); % number indicates the state in order (C1 C2 C3 C4 O5 O6)

cm=c(end)*ones(Num_IPR,1);

dt0=1e-3; % initial time stepsize in unit of second

dt=dt0;

g=zeros(Num_IPR,1); % tracking variable for determining transition time

r1=rand(Num_IPR,1); % uniform random variable on [0,1]

% IP3R parameters

q45=0;q54=3330;q12=1240;q21=0;q23=0;q32=69;q26=0;q62=4010;

% (Note that some parameters are set to be zero)

p=0.15; % muM

V42=110*p.^2./(p.^2+0.1^2);

k42=0.49+0.543*p.^3./(p.^3+4^3);

n42=3;

kn42=0.41+25*p.^3./(p.^3+6.5^3);

nn42=3;

a42=1.8*p.^2./(p.^2+0.58^2);

V24=62+880./(p.^2+4);

k24=0.35;

n24=3;

kn24=80;

nn24=2;

a24=1+5./(p.^2+0.5^2);

scale=4010/(4010+10500); % scaling factor due to model reduction (see main text for details)

% other model parameters (see main text for details)

Vs=10;

Ks=0.26;

kipr=0.05/Num_IPR*(1-scale);

kleak=0.0032;

kdiff=10;

Vp=0.8;

Kp=0.5;

Jleakin=0.03115;

Vrocc=0.2;

Jrocc=Vrocc*p;

Vsocc=1.6;

Ksocc=100;

h24_inf=kn24^nn24./(kn24^nn24+c0.^nn24);

h24=h24_inf*ones(Num_IPR,1);

h24_new=h24;

lambda_h24=40;

m24_inf=c0.^n24./(k24^n24+c0.^n24);

m24=m24_inf*ones(Num_IPR,1);

m24_new=m24;

lambda_m24=100;

m42_inf=c0.^n42./(k42.^n42+c0.^n42);

m42=m42_inf*ones(Num_IPR,1);

m42_new=m42;

lambda_m42=100;

h42_inf=kn42^nn42./(kn42^nn42+c0.^nn42);

h42=h42_inf*ones(Num_IPR,1);

h42_new=h42;

h42_track=h42;

ah42=0.5;

Vh42=20;

IT=1; % incremental time per iteration (s)

Numtimes=400; % number of iterations

% In the following, I will generate Numtimes files, each of which contain

% information connected in time. At last, I combine all the files in order

% to generate one file for analysis

wb=waitbar(0,'please wait...'); % show waitbar to indicate progress

for full=1:Numtimes

tic

Tmax=IT*full;

while time(end)<Tmax

No=0; % No is the number of open channels

for openind=1:Num_IPR

if state(openind,end)==2

No=No+1;

end

end

% First using the preceding time step dt to update the system (initial estimation)

Y0=[c(end);cb(end);ct(end)];

% here use RK4 to solve the ODEs

cs=gamma2*(Y0(3)-Y0(2)/gamma1-Y0(1));

Jdiff=kdiff*(Y0(2)-Y0(1));

Jleak=kleak*(cs-Y0(1));

Jserca=Vs*Y0(1)^1.75/(Y0(1)^1.75+Ks^1.75);

Jsocc=Vsocc*Ksocc^4/(cs^4+Ksocc^4);

Jin=Jleakin+Jrocc+Jsocc;

Jpm=Vp*Y0(1).^2./(0.5^2+Y0(1).^2);

Jipr=kipr*No*(cs-Y0(2));

YK1=dt*[Jdiff+Jleak-Jserca+Jin-Jpm

gamma1*(Jipr-Jdiff)

Jin-Jpm];

Y1=Y0+YK1/2;

cs=gamma2*(Y1(3)-Y1(2)/gamma1-Y1(1));

Jdiff=kdiff*(Y1(2)-Y1(1));

Jleak=kleak*(cs-Y1(1));

Jserca=Vs*Y1(1)^1.75/(Y1(1)^1.75+Ks^1.75);

Jsocc=Vsocc*Ksocc^4/(cs^4+Ksocc^4);

Jin=Jleakin+Jrocc+Jsocc;

Jpm=Vp*Y1(1).^2./(0.5^2+Y1(1).^2);

Jipr=kipr*No*(cs-Y1(2));

YK2=dt*[Jdiff+Jleak-Jserca+Jin-Jpm

gamma1*(Jipr-Jdiff)

Jin-Jpm];

Y2=Y0+YK2/2;

cs=gamma2*(Y2(3)-Y2(2)/gamma1-Y2(1));

Jdiff=kdiff*(Y2(2)-Y2(1));

Jleak=kleak*(cs-Y2(1));

Jserca=Vs*Y2(1)^1.75/(Y2(1)^1.75+Ks^1.75);

Jsocc=Vsocc*Ksocc^4/(cs^4+Ksocc^4);

Jin=Jleakin+Jrocc+Jsocc;

Jpm=Vp*Y2(1).^2./(0.5^2+Y2(1).^2);

Jipr=kipr*No*(cs-Y2(2));

YK3=dt*[Jdiff+Jleak-Jserca+Jin-Jpm

gamma1*(Jipr-Jdiff)

Jin-Jpm];

Y3=Y0+YK3;

cs=gamma2*(Y3(3)-Y3(2)/gamma1-Y3(1));

Jdiff=kdiff*(Y3(2)-Y3(1));

Jleak=kleak*(cs-Y3(1));

Jserca=Vs*Y3(1)^1.75/(Y3(1)^1.75+Ks^1.75);

Jsocc=Vsocc*Ksocc^4/(cs^4+Ksocc^4);

Jin=Jleakin+Jrocc+Jsocc;

Jpm=Vp*Y3(1).^2./(0.5^2+Y3(1).^2);

Jipr=kipr*No*(cs-Y3(2));

YK4=dt*[Jdiff+Jleak-Jserca+Jin-Jpm

gamma1*(Jipr-Jdiff)

Jin-Jpm];

Y_new=Y0+(YK1+2*YK2+2*YK3+YK4)/6;

c_new=Y_new(1);

cb_new=Y_new(2);

ct_new=Y_new(3);

dt1=dt*ones(Num_IPR,1);

% update gating variables

for i=1:Num_IPR

cs=gamma2*(ct_new(end)-cb_new(end)/gamma1-c_new(end));

cm(i)=cb_new*heaviside(state(i,end)-3)+120*(cs/100)*heaviside(3-state(i,end));

% updating m42

m42_inf=cm(i).^n42./(k42.^n42+cm(i).^n42);

YK1=dt*lambda_m42*(m42_inf-m42(i));

Y1=m42(i)+YK1/2;

YK2=dt*lambda_m42*(m42_inf-Y1);

Y2=m42(i)+YK2/2;

YK3=dt*lambda_m42*(m42_inf-Y2);

Y3=m42(i)+YK3;

YK4=dt*lambda_m42*(m42_inf-Y3);

m42_new(i)=m42(i)+(YK1+2*YK2+2*YK3+YK4)/6;

% updating h42

h42_inf=kn42^nn42./(kn42^nn42+cm(i).^nn42);

lambda_h42=ah42*heaviside(state(i,end)-3)+Vh42*heaviside(3-state(i,end));

YK1=dt*lambda_h42*(h42_inf-h42(i));

Y1=h42(i)+YK1/2;

YK2=dt*lambda_h42*(h42_inf-Y1);

Y2=h42(i)+YK2/2;

YK3=dt*lambda_h42*(h42_inf-Y2);

Y3=h42(i)+YK3;

YK4=dt*lambda_h42*(h42_inf-Y3);

h42_new(i)=h42(i)+(YK1+2*YK2+2*YK3+YK4)/6;

% updating m24

m24_inf=cm(i).^n24./(k24^n24+cm(i).^n24);

YK1=dt*lambda_m24*(m24_inf-m24(i));

Y1=m24(i)+YK1/2;

YK2=dt*lambda_m24*(m24_inf-Y1);

Y2=m24(i)+YK2/2;

YK3=dt*lambda_m24*(m24_inf-Y2);

Y3=m24(i)+YK3;

YK4=dt*lambda_m24*(m24_inf-Y3);

m24_new(i)=m24(i)+(YK1+2*YK2+2*YK3+YK4)/6;

% updating h24

h24_inf=kn24^nn24./(kn24^nn24+cm(i).^nn24);

YK1=dt*lambda_h24*(h24_inf-h24(i));

Y1=h24(i)+YK1/2;

YK2=dt*lambda_h24*(h24_inf-Y1);

Y2=h24(i)+YK2/2;

YK3=dt*lambda_h24*(h24_inf-Y2);

Y3=h24(i)+YK3;

YK4=dt*lambda_h24*(h24_inf-Y3);

h24_new(i)=h24(i)+(YK1+2*YK2+2*YK3+YK4)/6;

% the next step is to determine the transition time by linear interpolation

q24=(a24+V24*(1-m24(i).*h24(i)))*scale;

q42=a42+V42*m42(i).*h42(i);

Ta_old=[0 q12 0 0 0 0 ;q21 0 q23 q24 0 q26;0 q32 0 0 0 0;0 q42 0 0 q45 0;...

0 0 0 q54 0 0;0 q62 0 0 0 0]; % old transition matrix

q24=(a24+V24*(1-m24_new(i).*h24_new(i)))*scale;

q42=a42+V42*m42_new(i).*h42_new(i);

Ta_new=[0 q12 0 0 0 0 ;q21 0 q23 q24 0 q26;0 q32 0 0 0 0;0 q42 0 0 q45 0;...

0 0 0 q54 0 0;0 q62 0 0 0 0]; % new transition matrix

g_old=g(i);

g_new=g_old+(sum(Ta_old(state(i,end),:))+sum(Ta_new(state(i,end),:)))/2*dt; % trapezoid intergration

epsilon=log(1/r1(i));

if g_new>=epsilon % transition occurs

dt1(i)=(epsilon-g_old)/(g_new-g_old)*dt; % determine the transition time by linear interpolation

end

end

[dt_min index]=min(abs(dt1));

if index==1 && dt_min/dt==1

index=0;

end

dt=dt_min; % the found time interval from preceding time to transition time

% Now reupdate the system again using the "true" dt.

Y0=[c(end);cb(end);ct(end)];

% here use RK4 to solve the ODEs

cs=gamma2*(Y0(3)-Y0(2)/gamma1-Y0(1));

Jdiff=kdiff*(Y0(2)-Y0(1));

Jleak=kleak*(cs-Y0(1));

Jserca=Vs*Y0(1)^1.75/(Y0(1)^1.75+Ks^1.75);

Jsocc=Vsocc*Ksocc^4/(cs^4+Ksocc^4);

Jin=Jleakin+Jrocc+Jsocc;

Jpm=Vp*Y0(1).^2./(0.5^2+Y0(1).^2);

Jipr=kipr*No*(cs-Y0(2));

YK1=dt*[Jdiff+Jleak-Jserca+Jin-Jpm

gamma1*(Jipr-Jdiff)

Jin-Jpm];

Y1=Y0+YK1/2;

cs=gamma2*(Y1(3)-Y1(2)/gamma1-Y1(1));

Jdiff=kdiff*(Y1(2)-Y1(1));

Jleak=kleak*(cs-Y1(1));

Jserca=Vs*Y1(1)^1.75/(Y1(1)^1.75+Ks^1.75);

Jsocc=Vsocc*Ksocc^4/(cs^4+Ksocc^4);

Jin=Jleakin+Jrocc+Jsocc;

Jpm=Vp*Y1(1).^2./(0.5^2+Y1(1).^2);

Jipr=kipr*No*(cs-Y1(2));

YK2=dt*[Jdiff+Jleak-Jserca+Jin-Jpm

gamma1*(Jipr-Jdiff)

Jin-Jpm];

Y2=Y0+YK2/2;

cs=gamma2*(Y2(3)-Y2(2)/gamma1-Y2(1));

Jdiff=kdiff*(Y2(2)-Y2(1));

Jleak=kleak*(cs-Y2(1));

Jserca=Vs*Y2(1)^1.75/(Y2(1)^1.75+Ks^1.75);

Jsocc=Vsocc*Ksocc^4/(cs^4+Ksocc^4);

Jin=Jleakin+Jrocc+Jsocc;

Jpm=Vp*Y2(1).^2./(0.5^2+Y2(1).^2);

Jipr=kipr*No*(cs-Y2(2));

YK3=dt*[Jdiff+Jleak-Jserca+Jin-Jpm

gamma1*(Jipr-Jdiff)

Jin-Jpm];

Y3=Y0+YK3;

cs=gamma2*(Y3(3)-Y3(2)/gamma1-Y3(1));

Jdiff=kdiff*(Y3(2)-Y3(1));

Jleak=kleak*(cs-Y3(1));

Jserca=Vs*Y3(1)^1.75/(Y3(1)^1.75+Ks^1.75);

Jsocc=Vsocc*Ksocc^4/(cs^4+Ksocc^4);

Jin=Jleakin+Jrocc+Jsocc;

Jpm=Vp*Y3(1).^2./(0.5^2+Y3(1).^2);

Jipr=kipr*No*(cs-Y3(2));

YK4=dt*[Jdiff+Jleak-Jserca+Jin-Jpm

gamma1*(Jipr-Jdiff)

Jin-Jpm];

Y_new=Y0+(YK1+2*YK2+2*YK3+YK4)/6;

c_new=Y_new(1);

cb_new=Y_new(2);

ct_new=Y_new(3);

for i=1:Num_IPR

cs=gamma2*(ct_new(end)-cb_new(end)/gamma1-c_new(end));

cm(i)=cb_new*heaviside(state(i,end)-3)+120*(cs/100)*heaviside(3-state(i,end));

% updating m42

m42_inf=cm(i).^n42./(k42.^n42+cm(i).^n42);

YK1=dt*lambda_m42*(m42_inf-m42(i));

Y1=m42(i)+YK1/2;

YK2=dt*lambda_m42*(m42_inf-Y1);

Y2=m42(i)+YK2/2;

YK3=dt*lambda_m42*(m42_inf-Y2);

Y3=m42(i)+YK3;

YK4=dt*lambda_m42*(m42_inf-Y3);

m42_new(i)=m42(i)+(YK1+2*YK2+2*YK3+YK4)/6;

% updating h42

h42_inf=kn42^nn42./(kn42^nn42+cm(i).^nn42);

lambda_h42=ah42*heaviside(state(i,end)-3)+Vh42*heaviside(3-state(i,end));

YK1=dt*lambda_h42*(h42_inf-h42(i));

Y1=h42(i)+YK1/2;

YK2=dt*lambda_h42*(h42_inf-Y1);

Y2=h42(i)+YK2/2;

YK3=dt*lambda_h42*(h42_inf-Y2);

Y3=h42(i)+YK3;

YK4=dt*lambda_h42*(h42_inf-Y3);

h42_new(i)=h42(i)+(YK1+2*YK2+2*YK3+YK4)/6;

% updating m24

m24_inf=cm(i).^n24./(k24^n24+cm(i).^n24);

YK1=dt*lambda_m24*(m24_inf-m24(i));

Y1=m24(i)+YK1/2;

YK2=dt*lambda_m24*(m24_inf-Y1);

Y2=m24(i)+YK2/2;

YK3=dt*lambda_m24*(m24_inf-Y2);

Y3=m24(i)+YK3;

YK4=dt*lambda_m24*(m24_inf-Y3);

m24_new(i)=m24(i)+(YK1+2*YK2+2*YK3+YK4)/6;

% updating h24

h24_inf=kn24^nn24./(kn24^nn24+cm(i).^nn24);

YK1=dt*lambda_h24*(h24_inf-h24(i));

Y1=h24(i)+YK1/2;

YK2=dt*lambda_h24*(h24_inf-Y1);

Y2=h24(i)+YK2/2;

YK3=dt*lambda_h24*(h24_inf-Y2);

Y3=h24(i)+YK3;

YK4=dt*lambda_h24*(h24_inf-Y3);

h24_new(i)=h24(i)+(YK1+2*YK2+2*YK3+YK4)/6;

q24=(a24+V24*(1-m24(i).*h24(i)))*scale;

q42=a42+V42*m42(i).*h42(i);

Ta_old=[0 q12 0 0 0 0;q21 0 q23 q24 0 q26;0 q32 0 0 0 0;0 q42 0 0 q45 0;...

0 0 0 q54 0 0;0 q62 0 0 0 0];

q24=(a24+V24*(1-m24_new(i).*h24_new(i)))*scale;

q42=a42+V42*m42_new(i).*h42_new(i);

Ta_new=[0 q12 0 0 0 0;q21 0 q23 q24 0 q26;0 q32 0 0 0 0;0 q42 0 0 q45 0;...

0 0 0 q54 0 0;0 q62 0 0 0 0];

g(i)=g(i)+(sum(Ta_old(state(i,end),:))+sum(Ta_new(state(i,end),:)))/2*dt; % update tracking variable

end

% add updated data to exsiting vectors

c=[c,c_new];

cb=[cb,cb_new];

ct=[ct,ct_new];

time=[time,time(end)+dt];

h24=h24_new;

m24=m24_new;

h42=h42_new;

h42_track=[h42_track,h42_new];

m42=m42_new;

% After finding the transition time and the channel that changes at the

% time, now I determine which state the channel should enter.

if index==0

state=[state,state(:,end)];

dt=dt0;

else

q24=(a24+V24*(1-m24(index).*h24(index)))*scale;

q42=a42+V42*m42(index).*h42(index);

Ta_new=[0 q12 0 0 0 0 ;q21 0 q23 q24 0 q26;0 q32 0 0 0 0;0 q42 0 0 q45 0;...

0 0 0 q54 0 0;0 q62 0 0 0 0];

r2=rand; % uniform random variable on [0,1]

previous=Ta_new(state(index,end),:);

kk=1;

while sum(previous(1:kk))/sum(previous)<r2

kk=kk+1;

end

current=state(:,end);

current(index)=kk;

state=[state,current];

r1(index)=rand; % reset r1

g(index)=0; % reset corresponding value of g back to zero

end

end

toc

% save each interation

save(['2state_Part_',num2str(1000*p),'_',num2str(full)])

% update the variables used in the next interation

time=time(end);

c=c(end);

cb=cb(end);

ct=ct(end);

state=state(:,end);

h42_track=h42_track(:,end);

waitbar((full)/Numtimes)

end

close(wb) % close waitbar

%% this code is for gathering all the files made by the above code to generate a full file

totaltime=[]; % time

Ca=[]; % c values

Cab=[]; % cb values

CaT=[]; % ct values

h42_total=[]; % h42

state_total=[]; % state vector

wb1=waitbar(0,'please wait...');

for ldf=1:Numtimes

load(['2state_Part_',num2str(1000*p),'_',num2str(ldf)])

% gathering all the data

totaltime=[totaltime,time];

Ca=[Ca,c];

Cab=[Cab,cb];

CaT=[CaT,ct];

h42_total=[h42_total,h42_track];

state_total=[state_total,state];

waitbar(ldf/Numtimes)

end

close(wb1)

% generate a MAT file for all the data

save(['Stochastic_model_2state_IP3R_model_IP3_',num2str(1000*p),'nM_total_',num2str(Numtimes),'s'])

%% this file is for reducing the file size by decreasing the data resolution

% the quality of the reduced data set is still excellent for statistical analysis

clear B B_new IT Jserca No Ta_new Ta_old Numtimes

clear Y0 Y1 Y2 Y3 YK1 YK2 YK3 YK4 Y_new

clear c cb c_new current cm dt1 dt_min epsilon full g g_new g_old

clear h24 h24_inf h24_new h42 h42_inf h42_new h42_track

clear m24 m24_inf m24_new m42 m42_inf m42_new

clear i index kk idf openind previous r1 r2 state time wb wb1

% decreasing resolution by a factor of 10.

c=Ca(1:10:end);

ct=CaT(1:10:end);

cb=Cab(1:10:end);

time=totaltime(1:10:end);

state=state_total(:,1:10:end);

h42=h42_total(:,1:10:end);

clear Ca Cab CaT totaltime state_total h42_total fluo

save(['Stochastic_model_2state_IP3R_model_IP3_',num2str(1000*p),'nM_total_',num2str(Numtimes),'s_smallversion'])
