# Supplementary material for: A Deterministic Model Predicts the Properties of Stochastic Calcium Oscillations in Airway Smooth Muscle Cells
Source: PLoS Comput Biol. 2014 Aug 14;10(8):e1003783. doi: 10.1371/journal.pcbi.1003783 (PMC4133161; doi:10.1371/journal.pcbi.1003783)
Supplement: Text S3 — Matlab code for experimental data analysis. (DOCX) [file pcbi.1003783.s004.docx]

%% data set index (bsl:baseline;Fmax:maximum of F/F0 trace)

% all the files are in a zip file as supporting information

% convert excel files to MAT files, then use the following codes.

% Or add a few commands to input data directly from the excel files to % Matlab.

% S1_SMC1_MCh200nM: 180-255s; bsl=1.3; Fmax=2.4

% S2_SMC3_MCh200nM: 135.5-211s; bsl=1.15; Fmax=2.3

% S2_SMC4_MCh200nM: 120-180s; bsl=1.2; Fmax=2.5

% S2_SMC6_MCh200nM: 107-210s; bsl=1.4; Fmax=2.3

% S2_SMC7_MCh200nM: 60-180s; bsl=1.25; Fmax=2.8

% S2_SMC9_MCh200nM: 109-200s; bsl=1.5; Fmax=3.4

% S4_SMC2_MCh100nM: 109-220s; bsl=1.2; Fmax=2

% S4_SMC3_MCh100nM: 0-120s; bsl=1; Fmax=2.2

% S4_SMC4_MCh200nM: 99-150s; bsl=1.1; Fmax=2

% S5_SMC1_MCh100nM: 200-300s; bsl=1.1; Fmax=2.1

% S5_SMC1_MCh200nM: 50-300s; bsl=1.1; Fmax=2.2

% S5_SMC2_MCh100nM: 70-140s; bsl=1.5; Fmax=2.6

% S5_SMC3_MCh200nM: 195-300s; bsl=1.05; Fmax=1.8

% S5_SMC4_MCh200nM: 150-300s; bsl=1.5; Fmax=2.8

% S9_SMC1_MCh50nM: 50-150s; bsl=1.2; Fmax=2.3

%% choose data to run

load S5_SMC2_MCh100nM.mat

%% using moving average to reduce noise (every k points)

% use this to reproduce Figure 2A by choosing S9_SMC1_MCh50nM.mat

k=3;

Asum=0;

for i=1:k

Asum=Asum+A(i:end-k+i,3);

end

A1=Asum/k;

time=A(1:end-k+1,1);

%% getting samples for ISI, spike duration and amplitude from the data (for Figure 2B)

ISI=[]; % interspike interval vector

SD=[]; % spike duration vector

F_am=[]; % spike amplitude vector

bsl=1.5; % baseline

Fmax=2.6; % maximum of spike peak values

Lthed=(Fmax-bsl)*0.2+bsl; % setting low threshold

Hthed=(Fmax-bsl)*0.5+bsl; % setting high threshold

A1=A1(700:1399,:); % change the first index according to chosen period

% e.g. 50 - 150s should be 500:1500

F=heaviside(A1-Lthed);

F1=F(2:end)-F(1:end-1);

tindex=1:length(F1);

Fs=tindex'.*heaviside(F1-0.1);

Fe=tindex'.*heaviside(-F1-0.1);

Fs(abs(Fs) <= 0.1) = [];

Fe(abs(Fe) <= 0.1) = [];

spikestart=[]; % spike start index vector

spikeend=[]; % spike end index vector

peakind=[]; % spike peak index vector

for i=1:(length(Fe))

part=[Fs(i):Fe(i)];

[peak npeak]=max(A1(part));

if peak > Hthed

cpeak=max(A1(part));

F_am=[F_am cpeak];

spikestart=[spikestart Fs(i)];

spikeend=[spikeend Fe(i)];

peakind=[peakind Fs(i)+npeak-1];

end

end

for i=1:length(spikeend)

SD=[SD time(spikeend(i))-time(spikestart(i))];

end

for i=1:(length(spikeend)-1)

ISI=[ISI time(spikestart(i+1))-time(spikeend(i))];

end

% show results

mean(ISI)

std(ISI)

length(ISI)

mean(SD)

std(SD)

length(SD)

mean(F_am)

std(F_am)

length(F_am)

%% dependence of ISI, spike duration and amplitude on MCh concentration (Figure 4B, D and F)

% data are chosen as follows:

% MCh=50nM

% S9_SMC1_MCh50nM: 50-150s; bsl=1.2; Fmax=2.3

% S1_SMC2_MCh50nM: 180-350s; bsl=1.28; Fmax=2.275

% S9_SMC2_MCh50nM: 100-220s; bsl=1; Fmax=1.65

% MCh=100nM

% S4_SMC2_MCh100nM: 109-220s; bsl=1.2; Fmax=2

% S5_SMC1_MCh100nM: 200-300s; bsl=1.1; Fmax=2.1

% S5_SMC2_MCh100nM: 70-140s; bsl=1.5; Fmax=2.6

% MCh=200nM

% S5_SMC4_MCh200nM: 150-300s; bsl=1.5; Fmax=2.8

% S5_SMC3_MCh200nM: 195-300s; bsl=1.05; Fmax=1.8

% S4_SMC4_MCh200nM: 99-150s; bsl=1.1; Fmax=2

% S2_SMC4_MCh200nM: 120-180s; bsl=1.2; Fmax=2.5

% using the above codes, we can obtain the statistics as follows

MCh=[0.05,0.1,0.2];

ISIm=[8.6455,0.887,1.1688];

ISIstd=[10.8853,0.7336,0.7442];

ISInum=[33,165,199];

SDm=[0.6778,0.765,0.624];

SDstd=[0.1987,0.44,0.224];

SDnum=[36,168,203];

AMm=[1.92,2.07,2.074];

AMstd=[0.2478,0.2875,0.351];

AMnum=[36,168,203];

%% histograms for ISI (Figure 4B)

set(gcf,'position',[200 200 600 480])

axes('position',[0.18 0.18 0.75 0.75])

set(gcf,'position',[200 200 600 480])

h=bar(ISIm,0.5,'linewidth',2);

set(h,'FaceColor',[0 0 0])

hold on

errorbar([1:3],ISIm,ISIstd./sqrt(ISInum),'.k','markersize',1,'linewidth',2)

set(gca,'linewidth',2);

set(gca,'ticklength',1.5*get(gca,'ticklength'));

set(gca,'fontsize',22)

set(gca,'xticklabel',{'0.05','0.1','0.2'})

ylabel('ISI (s)','fontsize',25)

xlabel('MCh (\muM)','fontsize',25)

set(gca,'ylim',[0 12])

box off

%% histograms for spike duration (Figure 4F)

set(gcf,'position',[200 200 600 480])

axes('position',[0.18 0.18 0.75 0.75])

set(gcf,'position',[200 200 600 480])

h=bar(SDm,0.5,'linewidth',2);

set(h,'FaceColor',[0 0 0])

hold on

errorbar([1:3],SDm,SDstd./sqrt(SDnum),'.k','markersize',1,'linewidth',2)

set(gca,'linewidth',2);

set(gca,'ticklength',1.5*get(gca,'ticklength'));

set(gca,'fontsize',22)

set(gca,'xticklabel',{'0.05','0.1','0.2'})

ylabel('spike duration (s)','fontsize',25)

xlabel('MCh (\muM)','fontsize',25)

set(gca,'ylim',[0 1.3])

box off

%% histograms for amplitude (figure 4D)

set(gcf,'position',[200 200 600 480])

axes('position',[0.18 0.18 0.75 0.75])

set(gcf,'position',[200 200 600 480])

h=bar(AMm,0.5,'linewidth',2);

set(h,'FaceColor',[0 0 0])

hold on

errorbar([1:3],AMm,AMstd./sqrt(AMnum),'.k','markersize',1,'linewidth',2)

set(gca,'linewidth',2);

set(gca,'ticklength',1.5*get(gca,'ticklength'));

set(gca,'fontsize',22)

set(gca,'xticklabel',{'0.05','0.1','0.2'})

ylabel('F/F_0 peak','fontsize',25)

xlabel('MCh (\muM)','fontsize',25)

set(gca,'ylim',[0 3])

box off
