# Supplementary material for: A Deterministic Model Predicts the Properties of Stochastic Calcium Oscillations in Airway Smooth Muscle Cells
Source: PLoS Comput Biol. 2014 Aug 14;10(8):e1003783. doi: 10.1371/journal.pcbi.1003783 (PMC4133161; doi:10.1371/journal.pcbi.1003783)
Supplement: Text S4 — Matlab code for simulation analysis. (DOCX) [file pcbi.1003783.s005.docx]

% This file is for getting samples for ISI, spike duration and amplitude from the simulated data

% this can be used for both 6-state and 2-state models

clear all

format long

ISI=[]; % interspike interval vector

SD=[]; % spike duration vector

c_am=[]; % spike amplitude vector

% You need to load one MAT file here for following sampling and analysis

% e.g. load('Stochastic_model_2state_IP3R_model_IP3_150nM_total_400s_smallversion.mat')

bsl=0.097; % baseline can vary and is obtained from the time course of simulated calcium trace

bsl=bsl/(bsl+0.17)*2.7;

Fr=c./(c+0.17)*2.7; % convert [Ca2+] to fluorescence ratio

Fmax=max(Fr); % maximum of spike peak values

thrdL=(Fmax-bsl)*0.2+bsl; % setting low threshold

thrdH=(Fmax-bsl)*0.5+bsl; % setting high threshold

F=heaviside(Fr-thrdL);

F1=F(2:end)-F(1:end-1);

tindex=1:length(F1);

Fs=tindex.*heaviside(F1-0.1);

Fe=tindex.*heaviside(-F1-0.1);

Fs(abs(Fs) <= 0.1) = [];

Fe(abs(Fe) <= 0.1) = [];

spikestart=[]; % spike start index vector

spikeend=[]; % spike end index vector

peakind=[]; % spike peak index vector

for i=1:(length(Fe))

part=[Fs(i):Fe(i)];

[peak npeak]=max(Fr(part));

if peak > thrdH

cpeak=max(c(part));

c_am=[c_am cpeak];

spikestart=[spikestart Fs(i)];

spikeend=[spikeend Fe(i)];

peakind=[peakind Fs(i)+npeak-1];

end

end

for i=1:length(spikeend)

SD=[SD time(spikeend(i))-time(spikestart(i))];

end

for i=1:(length(spikeend)-1)

ISI=[ISI time(spikestart(i+1))-time(spikeend(i))];

end

% show results

mean(ISI)

std(ISI)

length(ISI)

mean(SD)

std(SD)

length(SD)

mean(c_am)

std(c_am)

length(c_am)
